# Complementary methods assessing short and long-term prey of a marine top predator ‒ Application to the grey seal-fishery conflict in the Baltic Sea

**DOI:** 10.1371/journal.pone.0208694

**Published:** 2019-01-02

**Authors:** Malin Tverin, Rodrigo Esparza-Salas, Annika Strömberg, Patrik Tang, Iiris Kokkonen, Annika Herrero, Kaarina Kauhala, Olle Karlsson, Raisa Tiilikainen, Markus Vetemaa, Tuula Sinisalo, Reijo Käkelä, Karl Lundström

**Affiliations:** 1 Molecular and Integrative Biosciences Research Programme, Faculty of Biological and Environmental Sciences, University of Helsinki, Helsinki, Finland; 2 Department of Biology, University of Oulu, Oulu, Finland; 3 Department of Environmental Research and Monitoring, Swedish Museum of Natural History, Stockholm, Sweden; 4 Natural Resources Institute, Luke, Helsinki, Finland; 5 Natural Resources Institute, Luke, Turku, Finland; 6 Metsähallitus Parks & Wildlife, Savonlinna, Finland; 7 Estonian Marine Institute, University of Tartu, Tartu, Estonia; 8 Department of Biological and Environmental Science, University of Jyväskylä, Jyväskylä, Finland; 9 Department of Aquatic Resources, Swedish University of Agricultural Sciences, Lysekil, Sweden; University of Hong Kong, HONG KONG

## Abstract

The growing grey seal (*Halichoerus grypus*) population in the Baltic Sea has created conflicts with local fisheries, comparable to similar emerging problems worldwide. Adequate information on the foraging habits is a requirement for responsible management of the seal population. We investigated the applicability of available dietary assessment methods by comparing morphological analysis and DNA metabarcoding of gut contents (short-term diet; n = 129/125 seals, respectively), and tissue chemical markers *i*.*e*. fatty acid (FA) profiles of blubber and stable isotopes (SIs) of liver and muscle (mid- or long-term diet; n = 108 seals for the FA and SI markers). The methods provided complementary information. Short-term methods indicated prey species and revealed dietary differences between age groups and areas but for limited time period. In the central Baltic, herring was the main prey, while in the Gulf of Finland percid and cyprinid species together comprised the largest part of the diet. Perch was also an important prey in the western Baltic Proper. The DNA analysis provided firm identification of many prey species, which were neglected or identified only at species group level by morphological analysis. Liver SIs distinguished spatial foraging patterns and identified potentially migrated individuals, whereas blubber FAs distinguished individuals frequently utilizing certain types of prey. Tissue chemical markers of adult males suggested specialized feeding to certain areas and prey, which suggest that these individuals are especially prone to cause economic losses for fisheries. We recommend combined analyses of gut contents and tissue chemical markers as dietary monitoring methodology of aquatic top predators to support an optimal ecosystem-based management.

## Introduction

Increasing seal populations worldwide have created resource competition and conflicts between the seals and local commercial fisheries, leading to culling programs with uncertain benefits [1,2]. Thus, reliable scientific data on seal feeding habits and resource exploitation is required. The Baltic grey seal (*Halichoerus grypus*) population has recovered from the low numbers in the 1980s, caused by extensive hunting and environmental toxins, to about 30 000 counted animals [3,4]. Consequently, conflicts with coastal fisheries have increased, mainly due to damage to catch and fishing gear [5] but also because of possible resource competition and bycaught seals [6]. Selective removal of specialized problem seals has been suggested as a method to mitigate damage to fisheries and at the same time avoid overhunting [5,7].

Targeted hunt of problem seals is feasible if individual preferences to certain feeding areas and prey species exist. Baltic grey seals, as a population, have been considered opportunistic predators, an interpretation based on analysis of gut contents [8–10]. According to these studies, herring (*Clupea harengus*), is the most important prey, followed by cod (*Gadus morhua*) and sprat (*Sprattus sprattus*) in the Baltic Proper, and common whitefish (*Coregonus lavaretus*) and vendace (*Coregonus albula*) in the Gulf of Bothnia. However, it is not known how representative this information is. The traditional diet estimation method, based on morphological identification of the prey remains in the gut, only represents the most recent diet, and might be biased towards prey with long-retained hard parts (HP). Currently, the HP analysis could be complemented with DNA analysis of the gut contents which may reduce bias caused by digestive erosion [11] and reveal prey with no recognizable hard parts [12]. ICES geographical regions (subdivisions, SD) are commonly used to assess and manage fish stocks in the Baltic Sea, and these regions correspond to spatial differences in hydrography and ecology [13]. Since previous studies utilizing HP analysis have identified ICES geographical regions, sampling gear type and age group as the most important explanatory factors for Baltic grey seal diet variation [8], the seals sampled for this study were grouped accordingly. In addition, possible ecological differences between the western and eastern coast of the same ICES SD were also taken into account when grouping the individuals. The effect of gender on the diet has been regarded as less important factor [8], although dietary differences between male and female grey seals have been documented in other areas [14].

Recent studies have suggested that individual grey seals, instead of being opportunistic, have specialized feeding areas and behaviours [5,15,16]. Although Baltic grey seals are capable of long-distance movements, even between ICES subdivisions, available information suggests that they forage on a more local spatial scale in the vicinity of preferred haul-out areas, however with substantial individual differences [16]. The possible fidelity of the individuals for certain foraging area was addressed by recording the locations and gear types the individuals were found in. Provided that the individuals from the same area and gear-type systematically show similar dietary marker profiles, which however are different from the marker profiles of the individuals from other gear types within possible daily range of swimming, the seals likely have settled feeding areas and habits. Methods providing estimates on long-term diet may help to reveal such individual specialization in certain feeding areas and types of prey consumed therein. This long-term dietary information can be obtained from chemical markers in predator tissues, such as blubber, liver and muscle. Transfer of dietary fatty acids (FAs) into marine mammal blubber is assumed to occur with little metabolic remodeling, which makes the FAs suitable for diet monitoring [17,18]. However, when using blubber FAs to study seal feeding ecology it should be noted that seal blubber is vertically layered and the composition of the outermost layer is fairly stable due to its thermoregulatory role [19‒21]. The middle and innermost layers are regarded metabolically active, with the inner layer assumingly reflecting mid-term (a few weeks) diet, whereas the middle layer integrates long-term (several months) dietary information [20,22]. Differing fractionation of heavy (*e*.*g*. δ^13^C, δ^15^N or δ^34^S) and light element isotopes in prey leads to predictable changes in the stable isotope (SI) values in predator tissues and different tissues can provide SI-based dietary information in different time scales, *e*.*g*. weeks for liver samples and months for muscle samples [23,24]. To be successfully accomplished, the FA and SI analyses require extensive prey FA and SI libraries.

By using data from a variety of methods it is possible to get estimates on short-, mid- and long-term diets of individual seals. The first aim of the study was to compare the short-term diet estimates obtained from HP and DNA analysis of grey seal gut contents, and to investigate the complementarity of these two methods. Second, we compared the power of tissue FA and SI profiles in assessing mid- and long-term feeding habits and examined whether these methods are able to reveal individual, or age- and sex-group related specialization. We hypothesized that the results from HP and DNA analyses would differ from each other. Further, we hypothesized that significantly different chemical marker profiles refer to individuals specialized in a certain foraging area and/or diet, whereas similar marker profiles would mean no preferential use of habitat or prey. Growing seal populations may adopt new foraging areas and resources, and adequate information on the spatial and temporal dietary variability clarifies the ecological role of marine mammals and may offer means for mitigating conflicts between seals and fisheries. In addition, dietary shifts and tissue chemistry of top predators are integrated proxies of food web changes, thus indicating the dynamics and health of the ecosystem [25,26].

## Methods

### Sample collection

**Fig 1:**
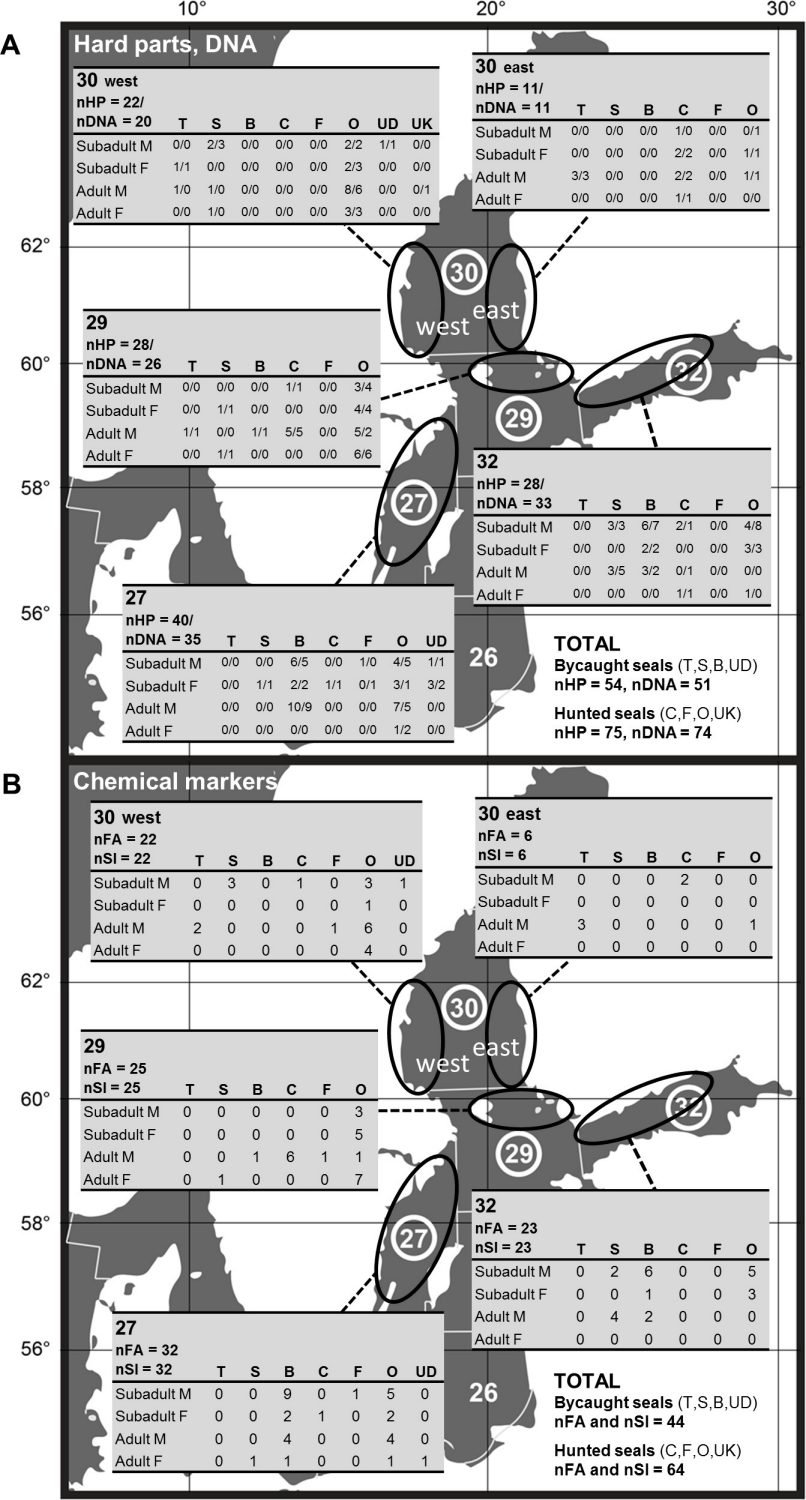
Sites of collection of the grey seals studied for the dietary proxies. ICES areas of the Baltic Sea and number of different grey seal individuals collected from the subdivisions 27, 29 (only the north-eastern part *i*.*e*. the archipelago between Åland Islands and Turku, was included), 30 (divided into SD30 west and SD30 east groups) and 32 (eastern part, the Russian sea area decluded), and studied for A) gut contents (HP and DNA, n = 129 and 125, respectively) and B) tissue chemical markers (blubber FAs and tissue SIs, n = 108). Seal individuals (M = male, F = female) were further categorized by the way/place of collection: T = trawl, S = surface fyke, B = bottom fyke, C = close to fishing gear, F = by fish farm, O = open water, UD = undefined fishing gear and UK = unknown hunting area (C/F/O inside the SD). Bycaught seals = T, S, B, UD; hunted seals C, F, O, UK.

The seal and fish samples were collected in collaboration with ongoing national and international monitoring programmes of fish and seals: in Sweden promoted by the Environmental Protection Agency (http://www.swedishepa.se) and the Agency for Marine and Water Management (http://www.havochvatten.se) and carried out by the University of Agricultural Sciences (http://www.slu.se) and Museum of Natural History (http://www.nrm.se); in Finland conducted by the Natural Resources Institute (http://www.luke.fi). The samples were collected during 2011 and 2012 and covered the ICES SDs 27, 29, 30 and 32 of the Baltic Sea (Fig 1).

### Seal samples

Blubber, muscle, liver (n = 108 for each) and gut samples (n = 129 and 125 for HP and DNA, respectively) from grey seals (all sample types were taken from 67 individuals) were collected during 2011 and 2012. The SD29 and 30 include pelagic and coastal areas, and the west and east coast ecosystems could provide the seals with different diet having distinct chemical markers. However, all the individuals from SD29 were collected in the archipelago between Åland Islands and Turku, and thus formed an ecologically uniform sample. The seals collected in SD 30 were mainly from the west coast (n = 22, except for DNA n = 20) but specimens of the east coast (n = 11 HP/DNA, n = 6 for FA/SI) were included as well, and thus the seals were subgrouped into western SD30 and eastern SD30. Seal sex and age (number of cementum zones in canine teeth longitudinal sections [27]) were recorded, as well as information on sampling location, date and cause of death: shot either close to fishing gear (C) or fish farm (F) or in other areas (O) or bycaugth with different gears. The type of fishing gear was documented: trawl (T), surface fyke (S) or bottom fyke (B). The surface fykes had the floating push-up design and were meant to catch the large pelagic species, Atlantic salmon (*Salmo salar*), sea trout (*Salmo trutta*) and common white fish, whereas the bottom fykes were placed at the bottom, had various traditional structures, and were meant to catch perch (*Perca fluviatilis*), pikeperch (*Sander lucioperca*), European eel (*Anguilla Anguilla*) and cyprinids. The gear type information was used in this study to define the specific habitat of the area where the seal was collected. Since 5 years is the most likely age of the first birth of the grey seal [28], the 0–4 year-old seals were classified as subadults and the 5+ year-old seals as adults. The hunted seal individuals (n = 75, 74, 64 for HP, DNA and FA/SI) were sampled in the field, and the digestive tract and original large-size tissue samples were stored in freezer (-25°C) before subsampled in the laboratory (for HP, DNA, FA and SI analysis). The bycaught seals (n = 54, 51, 44 for HP, DNA and FA/SI) were collected whole and stored in freezer (-25°C) before sampled and subsampled in the laboratory during autopsy. The sample storage time before the analyses of the material was less than 6 months for all types of analyses.

### Reference library of prey fish tissue

Whole fish were stored in freezer (-25°C) before homogenized and sampled in the laboratory for the 4 types of analyses. The fish tissue library created consisted of 26 species but the profound species-level analyses of the whole data with regional comparisons remain out of the scope of this study and will be published separately. This full fish material of 433 individuals were at first used to address the chemical marker variability of the Baltic fishes, and subsequently the 11 most probable prey species (for FA n = 233 and for SI n = 216) were chosen for the comparative analyses of this study (Tables A-D in S1 Table). The full data, however, were utilized to identify the individual FAs responsible for the largest interspecies variation and thus bringing with them dietary information into predator tissues. For prey-predator comparisons of the study, the FAs and SIs of 11 key prey fish species (more than 200 fishes), caught from the main habitats of the study area and reported to form the base of the grey seal diet [8], were analysed and the power of FAs and SIs to distinguish these pelagic (herring, sprat, Atlantic salmon and sea trout), coastal predatory (pikeperch, pike *Esox lucius* and perch) and demersal (common whitefish, eelpout *Zoarces viviparus* and roach *Rutilus rutilus*) fish was demonstrated. European eel, being a migrating species was not categorized into any aforementioned habitat.

### Gut content morphological analysis

The morphological HP analysis followed the methodology described by Lundström et al. [8,29]. Briefly, contents from stomachs and intestines were placed on a 0.5 mm sieve and a small portion of the produced liquid sample was collected and stored at -20°C for subsequent DNA analysis. Preserved prey specimens were identified and measured, followed by identification of sieved otoliths and other HPs by using reference collections (5 specimens of varying size for each species) and literature [30,31]. Sizes, numbers and biomass of prey items (mostly fish with only a few invertebrate *Saduria entomon* specimens) ingested per individual seal were estimated by considering all prey HPs, known relationships between otolith size and fish size, and compensating for digestive erosion of otoliths [29].

### DNA metabarcoding of gut contents

DNA was extracted individually for every stomach, intestine and colon content sample using a QIAmp DNA stool minikit (Qiagen N. V. Venlo, Netherlands) following the manufacturer’s “protocol for Human DNA”. An approximately 270 base-pair long fragment of the 16s rDNA gene (hereby 16s) was amplified by polymerase chain reaction (PCR) to be used as a “DNA barcoding” marker for prey species identification. PCR primers, forward primer 16sPreyF (5’-CGTGCRAAGGTAGCG-3’) and reverse primer 16sPreyR (5’-CCTYGGGCGCCCCAAC-3’) were designed by aligning and identifying variable sections of 16s sequences from various marine vertebrates present in the Baltic Sea, including seals and aquatic birds. The 3’ nucleotide of the forward primer mismatches the 16s sequence of seals, which inhibits the amplification of seal DNA, maximizing the prey DNA amplification.

The primer pair was tested initially using reference DNA template from 47 different fish species and eight bird species from the Baltic Sea region. With the exception of Agnatha species (*Lampetra fluviatilis* and *Petromyzon marinus*), all samples produced equally strong PCR products as visualized in agarose gels (data not shown). Eight forward and eight reverse primers were synthesized containing unique combinations of six nucleotides at the 5’ end. Such primers were used to produce 64 unique “barcode” identifier combinations to facilitate multiplexing of individuals in parallel sequencing and subsequent de-multiplexing of the output data, as described by [32].

PCR reactions were carried out in volumes of 25 μL containing 12.5 μL HotStart *Taq* master mix (Qiagen), 1 μL of each PCR primer (10 μM concentration), and 2 μL or DNA extract. Cycling conditions included an initial 5 minute (min) denaturing step at 95°C; 40 cycles of denaturing at 94°C for 30 seconds (s), 54°C for 30 s and 68°C for 60 s; and ending with a final extension step of 72°C for 10 min.

PCR products were pooled in groups of 64 barcoded individuals. Pooled reactions were then used to construct DNA libraries for sequencing following the “Rapid library preparation method manual” for GS junior Titanium series (Roche, March 2012) with the following modifications: the nebulization step was omitted, the RLdNTP, RL T4 polymerase and RL Taq polymerase were not included in the fragment end-repair reaction, and the small fragment removal was carried out by agarose-gel size selection and excision. Each of the pooled 64 individual reaction libraries was prepared using a different molecular identifier adapter (MID). DNA libraries were sequenced in two different runs in a GS-Junior instrument (Roche), following the emPCR amplification manual- Lib-L” and the “Sequencing method manual GS junior Titanium Series” protocols (Roche).

The DNA sequence data output in FastA format and its respective quality scores were combined into a FastQ file using Galaxy [33]. Sequence reads with either a < 60 bp length, a quality score of < 15 or a non-defined base call (N-bases) of > 2% were filtered out from the dataset using PRINSEQ [34]. The sorting of the sequencing output file into individual libraries and individuals within libraries, respectively, was carried out using the program 454 tag sorting by Johan Nylander (https://github.com/nylander/454_tag_sorting). Comparisons were performed using the BLASTn algorithm and species identification from the output sequences was carried out using the BLAST+ program [35], with the individually tagged DNA sequences (in FastA format) as a query database, and the nucleotide collection (nr) as a reference subject database. Only the highest score of each comparison was kept. Matching database records were then compared individually using BLASTn in order to identify and correct ambiguous matches. Individual samples that produced less than 100 valid prey sequence matched were discarded from further analyses. Finally, the dietary data from the DNA analysis were expressed as relative proportions of taxon specific sequences within a sample. The proportion of DNA sequences from a prey species indicates its contribution to the diet but it is not equal to the relative biomass consumed [36,37].

### Seal blubber and fish fatty acid analysis

Blubber samples were consistently collected from above sternum. In addition, the accurate sampling location has been reported to have negligible influence on the FA composition of pinniped blubber [20]. The blubber and reference fish samples were stored at—20°C until analysis. FA methyl esters were prepared from subsamples of blubber (dissected with skin and muscle) according to published protocols [20] and homogenates of whole fish (2 g subsample). Upon sampling, the blubber was frozen in liquid nitrogen, and vertically adjacent subsamples were taken by 3 mm intervals from skin to muscle, where the 3–6 mm above muscle represented the inner layer and the 6 mm above muscle to 18 mm below skin represented the middle layer. These boundaries for the middle layer were confirmed by studying the complete vertical profiles for each FA mol% in the blubber column of the adult males. Similar layers with same FA characteristics were found in the grey seals of this study as reported previously for ringed seals [20,21].

The FA composition in the seal and fish tissue samples was analyzed by gas chromatography according to previously published procedures [20,38] using a Shimadzu GC-2010 Plus equipment (Shimadzu Scientific Instruments, Kyoto, Japan) with flame-ionization detector (FID) for quantification of the FAs. Identification of the FA structures was performed by Shimadzu GCMS-QP2010 Ultra (Shimadzu) with mass selective detector (MSD). Both systems were equipped with Zebron ZB-wax capillary columns (30 m, 0.25 mm ID and film thickness 0.25 μm; Phenomenex, Torrence CA, USA). The FA compositions were expressed as mol% profiles, and the FAs were abbreviated: [carbon number]:[number of double bonds] n-[position of the first double bond calculated from the methyl end] (*e*.*g*. 20:5n-3). When studying the fish homogenates (of 26 Baltic species), 9 FAs (14:0, 16:1n-7, 18:1n-9, 18:2n-6, 18:3n-3, 18:4n-3, 20:1n-7, 20:4n-6, 22:6n-3) explained the most part of the interspecific variation and thus these were used as dietary markers for the seals. These FAs showed the largest relative standard deviations among the FAs present with levels not affected by methodological variation (only the FAs with signals exceeding 10x the replicate variation level were accepted for marker candidates), and they also were responsible for the main part of the data variation in the Principal Component Analysis (PCA; see Statistics) using as loadings standardized mol% data of either the full 26 species or the selected 11 main prey species.

### Seal tissue and fish stable isotope analysis

Seal muscle and liver samples, and the reference fish homogenates were frozen, freeze-dried and powdered for δ^13^C, δ^15^N and δ^34^S analyses. A maximum of 0.6 mg of each sample was loaded into a 4x6 mm tin capsule and combusted in Elementar Vario Pyrocube elemental analyser (Elementar, Germany) connected to Isoprime 100 CF-IRMS (Isoprime UK) mass spectrometer. Differences in the isotope values were measured relative to standards and expressed as per mil (‰) deviation from Vienna PeeDee belemnite (VPDB) for carbon, from atmospheric N_2_ (AIR) for nitrogen, and Vienna Canon Diablo Meteorite Troilite (V-CDT) for sulphur [39,40]. More precisely δ^13^C, δ^15^N, δ^34^S (‰) = (R sample / R standard– 1) × 103, where R sample is the ratio between the heavy isotope and its lighter counterpart for the sample, and R standard is the ratio for the international standard [41].

For standard reference materials pike muscle (FSS) standard was used as the internal laboratory standard, calibrated against isotopic standards (*e*.*g*. CH_6_, N_2_ and sphalerite NBS 123) provided by the International Atomic Energy Agency (IAEA, Vienna). FSS has known values of δ^13^Cstd = -26.39 ‰, δ^15^Nstd = 13.08 ‰ and δ^34^Sstd = 12.45 ‰ and was used as working standard to examine isotopic drift within and throughout the run. Elemental analysis standard reference material, sulfanilamide (IVA Analysentechnike. K.) was used to correct the % C, % N and % S data. As lipids are known to be ^13^C-depleted (having lower δ^13^C relative to other major tissue constituents as proteins) [42,43], carbon isotope values (δ^13^C) were ‘lipid normalized’ for both seal tissues and all fish samples using the C/N ratio according to [44].

### Turnover rates of tissue chemical markers

Information on the turnover rates of SIs and FAs in the studied seal tissues (liver, muscle, blubber) [20,45,46] suggests that the long-term dietary markers may further fall into two timescale categories. Regarding stable isotopes, seal liver has relatively fast turnover of biomolecules while the turnover of muscle is relatively slow [47‒50]. In mammals, the carbon turnover time in liver has a half-life of 6.4 days and muscle 27.6 days [47,48]. Some signal of past diet may still be detectable after a period roughly 2 to 3 times that of the isotopic half-life of the tissue [49,50]. Therefore, we assumed the δ^13^C, δ^15^N and δ^34^S values from seal liver reflect the dietary elements 2–3 weeks prior to sampling, and the isotope values from muscle tissue should represent the elements assimilated up to 2–3 months prior to sampling.

In mammals, clearance of circulating chylomicrons and absorption of FAs begins in min scale and the lipids not immediately needed for energy metabolism are stored in the adipose tissue [51,52]. This suggest that the FA composition in the innermost layers of blubber is likely affected after a brief postprandial period. Unfortunately, the systematic works defining the FA turnover rates at different depths of seal blubber are missing. Since dietary polyunsaturated FAs (PUFAs) are preferentially found in the inner layers of blubber, this layer is likely the metabolically most active layer of blubber [20,45]. This view is further supported by the facts that in ringed seals the innermost blubber shows the largest compositional similarities with the potential prey fish FAs, and that in accidentally caught individuals from the same area of Lake Saimaa the FA composition of the innermost blubber layers showed the largest individual compositional variability [20,45]. By using full layer biopsies in a harbour seal feeding experiment, Nordstrom et al. [53] estimated the overall blubber FA turnover rate being 2–3 months, thus giving a justified estimate for the middle blubber. The turnover rate of the FAs in the innermost blubber is likely much shorter.

### Statistics

FA and SI data were subjected to multivariate PCA (Sirius 8.5 software, Pattern Recognition Systems, Bergen, Norway) to assess compositional differences between the samples and highlight the marker FAs and SIs mainly responsible for the variation in the data. Prior to the analysis, FA data were *arcsine* (of the square root) transformed to improve data normality, and all FA and SI variables were standardized to prevent large components from dominating the analysis. Since systematic small differences in the relative concentrations of diet-reflecting small components of the FA profile may carry equally important dietary information as the differences in large components [54], the standardization procedure, despite losing the original ratios of the different FAs, was regarded as a sound choice. In PCA, the relative positions of the samples and variables were plotted using the first two principal components and separations between sample groups were tested for statistical significance by using Soft Independent Modelling of Class Analogy (SIMCA) [55,56] and regarding *P* < 0.05 significant. SIMCA is a supervised classification method building multiple PCA-based class models, and as a “soft” method it can classify a sample into several overlapping classes. SIMCA uses F-test to evaluate the sample Euclidean distances from the models, and it is regarded as a robust method, which can be applied to data having non-normal distribution, although it performs ideally with data having normal distribution or transformed for better normality [57]. However, when the limited data of 11 adult grey seal males (the ones with accurately recorded background information) were analyzed for chemical markers, we in parallel carried out the non-metric multidimensional scaling, nMDS (Primer 6, PRIMER-E, Auckland, New Zealand) and analysis of similarities, ANOSIM with non-transformed data in order to examine whether the results of these statistical analyses were sensitive to the type of multivariate method chosen.

## Results

### Indicators of short-term diet

In the diet of subadult males, morphological HP analysis (n = 37) and DNA metabarcoding (n = 42) identified similar number of prey taxa (15 species + 7 taxa versus 18 species + 5 taxa, respectively) (Table 1). Both methods identified herring (46.7% of the consumed mass as assessed from the HP analysis vs 39.8% of the DNA sequences), perch (13.6 vs 11.0%) and eelpout (10.3 vs 6.1%) as the most important prey species. DNA analysis indicated a markedly higher contribution of sprat, three-spined stickleback *Gasterosteus aculeatus* and cod to the diet compared to HP analysis (2.6 vs 9.2%, < 0.1 vs 5.3% and 0.2 vs 3.3%, respectively). DNA metabarcoding also detected the presence of important dietary species not identified by HP analysis: bream *Abramis brama* (6.0%) and burbot *Lota lota* (3.4%), and other 5 species with a DNA sequence proportion < 1% (sand goby *Pomatochistus minutus*, Atlantic salmon, white bream *Blicca bjoerkna*, black goby *Gobius niger* and rainbow trout *Onchorynchus mykiss*, in the order of descending proportion). The isopod cructacean *Saduria entomon* was detected by HP analysis (2.9%) but not by DNA analysis.

**Table 1:** Prey items of subadult and adult grey seal males indicated by gut morphological *i*.*e*. hard part (HP, mass %) and DNA (sequence %) prey proportions, and the frequencies of occurrence (Freq %).

Prey items	Subadult males				Adult males			
	HP (%) n = 37		DNA (%) n = 42		HP (%) n = 51		DNA (%) n = 44	
	Prop	Freq	Prop	Freq	Prop	Freq	Prop	Freq
Baltic herring *Clupea harengus*	46.7	*59*.*5*	39.8	*69*.*0*	29.0	*58*.*8*	24.6	*63*.*6*
Perch *Perca fluviatilis*	13.6	*21*.*6*	11.0	*21*.*4*	13.0	*35*.*3*	11.1	*36*.*4*
Eelpout *Zoarces viviparus*	10.3	*13*.*5*	6.1	*28*.*6*	7.0	*17*.*6*	*8*.*5*	*18*.*2*
Bream *Abramis brama*	ND	*ND*	6.0	*16*.*7*	3.4	*5*.*9*	10.8	*22*.*7*
Roach *Rutilus rutilus*	3.0	*8*.*1*	3.4	*11*.*9*	5.0	*13*.*7*	5.1	*31*.*8*
Sprat *Sprattus sprattus*	2.6	*13*.*5*	9.2	*23*.*8*	0.5	*7*.*8*	2.9	*22*.*7*
Pikeperch *Sander lucioperca*	4.3	*5*.*4*	2.4	*16*.*7*	1.5	*7*.*8*	5.8	*13*.*6*
Common whitefish *C*. *lavaretus*	0.3	*2*.*7*	ND	*ND*	9.2	*21*.*6*	ND	*ND*
Atlantic salmon *Salmo salar*	ND	*ND*	0.6	*9*.*5*	1.5	*3*.*9*	6.2	*6*.*8*
European eel *Anguilla anguilla*	3.0	*5*.*4*	1.7	*7*.*1*	1.6	*3*.*9*	1.0	*6*.*8*
Three-spined stickleback *G*. *aculeatus*	< 0.1	*2*.*7*	5.3	*7*.*1*	ND	*ND*	<0.1	*2*.*3*
Cod *Gadus morhua*	0.2	*2*.*7*	3.3	*11*.*9*	1.1	*5*.*9*	0.3	*2*.*3*
Burbot *Lota lota*	ND	*ND*	3.4	*9*.*5*	0.5	*2*.*0*	0.5	*4*.*5*
Pike *Esox lucius*	ND	*ND*	ND	*ND*	1.8	*2*.*0*	2.2	*6*.*8*
Isopod crustacean *Saduria entomon*	2.9	*8*.*1*	ND	*ND*	<0.1	*3*.*9*	ND	*ND*
Sea trout *Salmo trutta*	0.9	*2*.*7*	ND	*ND*	<0.1	*2*.*0*	1.2	*2*.*3*
Ruffe *Gymnocephalus cernua*	1.0	*5*.*4*	0.3	*4*.*8*	<0.1	*2*.*0*	0.8	*2*.*3*
European flounder *Platichthys flesus*	ND	*ND*	ND	*ND*	1.7	*5*.*9*	ND	*ND*
Turbot *Scophthalmus maximus*	ND	*ND*	ND	*ND*	1.4	*2*.*0*	ND	*ND*
Four-horned sculpin *M*. *quadricornis*	0.4	*2*.*7*	ND	*ND*	0.9	*5*.*9*	ND	*ND*
Smelt *Osmerus eperlanus*	0.5	*8*.*1*	< 0.1	*2*.*4*	0.3	*5*.*9*	0.2	*6*.*8*
Sand goby *Pomatoschistus minutus*	ND	*ND*	0.7	*2*.*4*	ND	*ND*	< 0.1	*4*.*5*
Common dab *Limanda limanda*	ND	*ND*	ND	*ND*	0.7	*2*.*0*	ND	*ND*
White bream *Blicca bjoerkna*	ND	*ND*	< 0.1	*4*.*8*	ND	*ND*	0.2	*4*.*5*
Tench *Tinca tinca*	ND	*ND*	ND	*ND*	0.1	*2*.*0*	ND	*ND*
Black goby *Gobius niger*	ND	*ND*	< 0.1	*2*.*4*	<0.1	*2*.*0*	ND	*ND*
Rainbow trout *Oncorhynchus mykiss*	ND	*ND*	< 0.1	*2*.*4*	ND	*ND*	ND	*ND*
Cyprinids Cyprinidae	5.0	*16*.*2*	ND	*ND*	14.4	*31*.*4*	ND	*ND*
Whitefishes *Coregonus* spp	ND	*ND*	2.2	*11*.*9*	0.3	*3*.*9*	13.2	*27*.*3*
Percids Percidae	2.2	*5*.*4*	ND	*ND*	3.1	*11*.*8*	ND	*ND*
Sculpins Cottidae	< 0.1	*2*.*7*	2.4	*4*.*8*	<0.1	*3*.*9*	1.8	*13*.*6*
European flounder or plaice *Plat*. *flesus* or *Pleur*. *platessa*	ND	*ND*	< 0.1	*2*.*4*	ND	*ND*	2.4	*4*.*5*
Clupeids Clupeidae	0.3	*2*.*7*	ND	*ND*	2.0	*5*.*9*	ND	*ND*
Daces *Leuciscus* sp.	ND	*ND*	1.2	*2*.*4*	ND	*ND*	1.0	*2*.*3*
Sand lances Ammodytidae	1.1	*8*.*1*	0.7	*4*.*8*	ND	*ND*	ND	*ND*
Lampreys Petromyzontidae	1.7	*2*.*7*	ND	*ND*	ND	*ND*	ND	*ND*
Gobies Gobiidae	< 0.1	*5*.*4*	ND	*ND*	<0.1	*15*.*7*	ND	*ND*

The items identified at species levels were listed first (in the order of decreasing average proportion in the subadult and adult diets, indicated by the HP and DNA analyses) and those identified at species group levels were listed after the species. The number of subadult and adult males from which both HP and DNA were recorded was 33 and 40, respectively.

In the diet of adult males, morphological analysis (n = 51) distinguished higher number of prey taxa than the DNA analysis (n = 44) (23 species + 6 taxa vs 18 species + 4 taxa) (Table 1). Both methods identified herring (HP 29.0% vs DNA 24.6%) as an important prey. Cyprinids were also among the main items and the HP analysis estimated the proportion of roach and bream to 5.0 and 3.4%, respectively, and other undefined cyprinids to 14.4%. DNA metabarcoding increased the taxonomic resolution of cyprinids in the diet, showing a share of 10.8% for bream, 5.1% for roach, 1.0% for daces *Leuciscus sp*. and 0.2% for white bream. In addition, HP analysis reported a mass proportion of 9.2% for common whitefish while 13.2% of the DNA sequences belonged to *Coregonus* species, *i*.*e*. common whitefish or vendace. The contribution of Atlantic salmon, pikeperch, sprat, sea trout and ruffe *Gymnocephalus cernua* differed markedly between the methods with larger proportions indicated by the DNA analysis (1.5 vs 6.2%, 1.5 vs 5.8%, 0.5 vs. 2.9%, < 0.1 vs. 1.2%, and < 0.1% vs. 0.8%, respectively). In the adult males, turbot *Scophthalmus maximus*, four-horned sculpin *Myoxocephalus quadricornis*, common dab *Limanda limanda*, tench *Tinca tinca*, black goby and the benthic isopod *Saduria entomon* were only detected by the morphological analysis (listed in the order of descending proportion).

The HP analysis of female subadults (n = 26) distinguished a slightly lower number of prey taxa (11 species + 4 taxa) than DNA analysis (n = 25; 14 species + 4 taxa) (Table 2). Both methods identified herring as the most important dietary species (54.0 vs 38.3%). The species totally missed in HP analyses were sand goby, Atlantic salmon and roach with DNA sequence proportions of 4.1, 3.3 and 1.3%, respectively. In addition, bream, pikeperch, rainbow trout and Cottidae species, *i*.*e*. sculpins were also only detected in the DNA analysis but with DNA sequence proportions < 1%.

**Table 2:** Prey items of subadult and adult grey seal females indicated by gut morphological *i*.*e*. hard part (HP, mass %) and DNA (sequence %) prey proportions, and the frequencies of occurrence (Freq %).

Prey items	Subadult females				Adult females			
	HP (%) n = 26		DNA (%) n = 25		HP (%) n = 15		DNA (%) n = 14	
	Prop	Freq	Prop	Freq	Prop	Freq	Prop	Freq
Baltic herring *Clupea harengus*	54.0	*80*.*8*	38.3	*76*.*0*	42.3	*80*.*0*	59.3	*85*.*7*
Eelpout *Zoarces viviparus*	9.1	*15*.*4*	15.4	*28*.*0*	23.2	*40*.*0*	20.0	*50*.*0*
Sprat *Sprattus sprattus*	11.4	*23*.*1*	12.6	*48*.*0*	ND	ND	1.1	*21*.*4*
Three-spined stickleback *G*. *aculeatus*	3.8	*3*.*8*	11.0	*20*.*0*	ND	ND	ND	ND
Cod *Gadus morhua*	3.8	*3*.*8*	5.7	*20*.*0*	1.0	*6*.*7*	3.2	*7*.*1*
Common whitefish *C*. *lavaretus*	0.4	*3*.*8*	ND	ND	12.8	*26*.*7*	ND	ND
Perch *Perca fluviatilis*	4.1	*7*.*7*	4.4	*24*.*0*	2.3	*13*.*3*	1.1	*7*.*1*
Black goby *Gobius niger*	ND	ND	ND	ND	1.9	*6*.*7*	2.8	*14*.*3*
Sand goby *Pomatoschistus minutus*	ND	ND	4.1	*16*.*0*	ND	ND	ND	ND
Atlantic salmon *Salmo salar*	ND	ND	3.3	*4*.*0*	ND	ND	ND	ND
Roach *Rutilus rutilus*	ND	*ND*	1.3	*4*.*0*	ND	*ND*	1.6	*14*.*3*
Vendace *Coregonus albula*	ND	ND	ND	ND	1.1	*6*.*7*	ND	ND
Bream *Abramis brama*	ND	ND	0.4	*12*.*0*	ND	ND	0.3	*21*.*4*
Ruffe *Gymnocephalus cernua*	< 0.1	*3*.*8*	0.6	*4*.*0*	ND	ND	ND	ND
Pikeperch *Sander lucioperca*	ND	ND	0.2	*8*.*0*	ND	ND	< 0.1	*14*.*3*
Smelt *Osmerus eperlanus*	< 0.1	*3*.*8*	0.1	*4*.*0*	< 0.1	*6*.*7*	ND	*ND*
European flounder *Platichthys flesus*	0.1	3.8	ND	ND	ND	ND	ND	ND
Rainbow trout *Oncorhynchus mykiss*	ND	ND	<0.1	*4*.*0*	ND	ND	ND	ND
Isopod crustacean *Saduria entomon*	< 0.1	*3*.*8*	ND	ND	ND	ND	ND	ND
Clupeids Clupeidae	4.9	*15*.*4*	ND	ND	6.7	*6*.*7*	ND	ND
Percids Percidae	7.2	*7*.*7*	ND	ND	0.8	*6*.*7*	ND	ND
Whitefishes *Coregonus* spp	ND	ND	0.3	*16*.*0*	ND	ND	7.6	*35*.*7*
Salmonids *Salmo* spp	ND	ND	ND	ND	5.9	*6*.*7*	ND	ND
Sculpins Cottidae	ND	ND	0.8	*4*.*0*	ND	ND	2.7	14.3
Gobies Gobiidae	0.6	*15*.*4*	ND	ND	2.1	*13*.*3*	ND	ND
European flounder or European plaice *Plat*. *flesus* or *Pleur*. *platessa*	ND	ND	1.1	*4*.*0*	ND	ND	< 0.1	*7*.*1*
Sand lances Ammodytidae	0.5	*7*.*7*	0.3	*8*.*0*	ND	ND	ND	ND

The items identified at species levels were listed first (in the order of decreasing average proportion in the subadult and adult diets, indicated by the HP and DNA analyses) and those identified at species group levels were listed after the species. The number of subadult and adult females from which both HP and DNA were recorded was 23 and 13, respectively.

Also in the adult females, there were variability in the taxa identified by the HP (n = 15; 8 species + 4 taxa) and DNA analysis (n = 14; 9 species + 3 taxa) but still the total number of taxa was the same (Table 2). Both methods identified herring (42.3 HP% vs 59.3 DNA%) as the most important dietary species, with eelpout (23.2 vs 20.0%), and common whitefish/*Coregonus* spp. (12.8 vs 7.6%) as other major items. The small contributions of roach, sprat, bream, pikeperch, flounder/plaice *Platichthys flesus/Pleuronectes platessa* and sculpins were only detected by the DNA analysis.

The short-term diets indicated by the HP and DNA analyses also differed between the Baltic ICES SDs (sufficient data were available for SD comparisons only for the males) (Tables 3 and 4). In general, herring clearly dominated the male diets in the Gulf of Bothnia (SD30) and remained one of the main dietary items in the Åland‒Turku archipelago (all samples from north-east SD29) and on the western coast of Baltic Proper (SD27), where perch was another common prey. In the Gulf of Finland (SD32) percids and cyprinids together became the main part of the diet.

In subadult males (Table 3), herring dominated the diet in all areas, followed by eelpout, perch, sprat and European eel in SD27; eelpout (and several species suggested important dietary constituents by either HP or DNA) in SD29; eelpout by HPs and cod by DNA in western SD30; perch, pikeperch and roach in SD32.

**Table 3:** Comparison of the prey of subadult grey seal males collected from four ICES subdivisions of the Baltic sea (27, 29, 30 and 32) indicated by gut morphological *i*.*e*. hard part (HP, mass %) and DNA (sequence %) prey proportions.

Prey of subadult males in ICES subdivisions	27	29	30 west*	32
	HP / DNA n = 12 / n = 11	HP / DNA n = 4 / n = 5	HP / DNA n = 5 / n = 6	HP / DNA n = 15 / n = 19
Baltic herring *Clupea harengus*	55.3 / 43.8	50.0 / 35.2	73.9 / 65.2	26.2 / 32.8
Eelpout *Zoarces viviparus*	10.5 / 12.3	24.9 / 6.3	12.7/ <0.1	6.1 / 4.4
Perch *Perca fluviatilis*	13.6 / 8.8	ND / ND	2.6 / 1.5	21.7 / 18.8
Sprat *Sprattus sprattus*	4.4 / 11.5	ND / 24.8	1.2 / ND	2.6 / 2.1
Three-spined stickleback *G*. *aculeatus*	ND / ND	0.1 / 33.7	ND / ND	ND / 2.9
Isopod crustacean *Saduria entomon*	ND / ND	25.1 / ND	ND / ND	0.4 / ND
Cod *Gadus morhua*	0.5 / 3.6	ND / ND	ND / 16.7	ND / ND
Pikeperch *Sander lucioperca*	ND / <0.1	ND / ND	ND / ND	10.6 / 5.3
European eel *Anguilla anguilla*	9.3 / 6.4	ND / ND	ND / <0.1	ND / ND
Bream *Abramis brama*	ND / 3.6	ND / 0.1	ND / ND	ND / 11.3
Roach *Rutilus rutilus*	ND / ND	ND / ND	ND / ND	7.3 / 7.5
Burbot *Lota lota*	ND / ND	ND / ND	ND / ND	ND / 7.6
Ruffe *Gymnocephalus cernua*	ND / ND	ND / ND	ND / ND	2.4 / 0.7
Sea trout *Salmo trutta*	2.8 / ND	ND / ND	ND / ND	ND / ND
Four-horned sculpin *M*. *quadricornis*	ND / ND	ND / ND	2.8 / ND	ND / ND
Atlantic salmon *Salmo salar*	ND / 2.4	ND / ND	ND / ND	ND / <0.1
Sand goby *Pomatoschistus minutus*	ND / ND	ND / ND	ND / ND	ND / 1.5
Smelt *Osmerus eperlanus*	ND / ND	ND / ND	ND / ND	1.3 / < 0.1
Common whitefish *C*. *lavaretus*	ND / ND	ND / ND	ND / ND	0.8 / ND
Rainbow trout *Oncorhynchus mykiss*	ND / <0.1	ND / ND	ND / ND	ND / ND
Black goby *Gobius niger*	ND / <0.1	ND / ND	ND / ND	ND / ND
White bream *Blicca bjoerkna*	ND / ND	ND / ND	ND / ND	ND / <0.1
Sculpins Cottidae	ND / ND	ND / ND	ND / 16.6	0.1 / < 0.1
Cyprinids Cyprinidae	2.1 / ND	ND / ND	ND / ND	10.8 / ND
Sand lances Ammodytidae	0.6 / 2.7	ND / ND	6.7 / ND	ND / ND
Percids Percidae	ND / ND	ND / ND	ND / ND	5.5 / ND
Whitefishes *Coregonus* spp	ND / 0.2	ND / ND	ND / ND	ND / 4.8
Daces *Leuciscus* sp.	ND / 4.5	ND / ND	ND / ND	ND / ND
Lampreys Petromyzontidae	ND / ND	ND / ND	ND / ND	4.1 / ND
Clupeids Clupeidae	0.9 / ND	ND / ND	ND / ND	ND / ND
Gobies Gobiidae	ND / ND	ND / ND	<0.1 / ND	0.2 / ND
European flounder or plaice *Plat*. *flesus* or *Pleur*. *platessa*	ND / 0.2	ND / ND	ND / ND	ND / ND

*Only two subadult males were collected from the eastern coast of SD30, the gut of one solely contained undefined herring/sprat by HP, and the prey of the other included 96.6% sprat and 3.4 mol% eelpout by DNA.

In adult males (Table 4), herring and perch were the most important short-term prey in SD27. In SD29, herring and cyprinids dominated the diet. In the western and eastern SD30, following the herring, large dietary proportions were detected for eelpout, perch and common whitefish (according to HPs). In SD32, cyprinids, perch, pikeperch, Atlantic salmon and sprat were important prey of the adult males.

**Table 4:** Comparison of the prey of adult grey seal males collected from four ICES subdivisions of the Baltic sea (27, 29, 30 and 32) indicated by gut morphological *i*.*e*. hard part (HP, mass %) and DNA (sequence %) prey proportions.

Prey of adult males in ICES subdivisions	27	29	30 west*	30 east	32
	HP / DNA n = 17 / n = 14	HP / DNA n = 12 / n = 9	HP / DNA n = 10 / n = 7	HP / DNA n = 6 / n = 6	HP / DNA n = 6 / n = 8
Baltic herring *Clupea harengus*	17.3 / 33.8	34.1 / 18.7	47.8 / 31.3	33.6 / 35.7	10.4 / 0.7
Perch *Perca fluviatilis*	21.6 / 24.9	5.0 / 2.0	7.9 / 12.1	9.4 / 5.7	16.2 / 0.1
Bream *Abramis brama*	ND / ND	12.1 / 34.3	ND / ND	ND / 1.3	4.9 / 19.7
Eelpout *Zoarces viviparus*	2.1 / <0.1	7.0 / 12.7	8.9 / 14.3	19.8 / 16.6	8.0 / 7.2
Roach *Rutilus rutilus*	9.3 / 8.7	2.4 / 8.8	ND / ND	ND / 0.4	11.7 / 3.0
Pikeperch *Sander lucioperca*	ND / ND	3.0 / 8.2	ND / ND	ND / ND	6.6 / 22.5
Atlantic salmon *Salmo salar*	ND / ND	ND / ND	7.5 / 12.2	ND / ND	ND / 23.3
Common whitefish *C*. *lavaretus*	12.2 / ND	2.7 / ND	14.3 / ND	17.9 / ND	ND / ND
Sprat *Sprattus sprattus*	1.3 / 0.2	ND / 0.5	0.1 / 0.2	ND / 3.4	0.3 / 12.4
Pike *Esox lucius*	5.4 / <0.1	ND / ND	ND / 4.6	ND / 10.9	ND / ND
European eel *Anguilla anguilla*	4.8 / 3.1	ND / ND	ND / <0.1	ND / ND	ND / ND
Sea trout *Salmo trutta*	ND / ND	ND / ND	0.5 / ND	ND / ND	ND / 6.7
Burbot *Lota lota*	ND / ND	ND / ND	ND / 3.4	ND / ND	4.4 / ND
Ruffe *Gymnocephalus cernua*	ND / ND	0.2 / 4.0	ND / ND	ND / ND	ND / ND
Cod *Gadus morhua*	3.2 / 1.1	ND / ND	ND / ND	ND / ND	ND / ND
Turbot *Scophthalmus maximus*	4.1 / ND	ND / ND	ND / ND	ND / ND	ND / ND
Four-horned sculpin *M*. *quadricornis*	ND / ND	0.4 / ND	ND / ND	8.2 / ND	ND / ND
Smelt *Osmerus eperlanus*	0.2 / 0.5	ND / <0.1	0.9 / ND	ND / ND	ND / ND
Common dab *Limanda limanda*	2.0 / ND	ND / ND	ND / ND	ND / ND	ND / ND
White bream *Blicca bjoerkna*	ND / 0.5	ND / ND	ND / ND	ND / ND	ND / ND
Sand goby *Pomatoschistus minutus*	ND / 0.2	ND / ND	ND / ND	ND / ND	ND / ND
Isopod crustacean *Saduria entomon*	<0.1 / ND	ND / ND	ND / ND	ND / ND	ND / ND
Black goby *Gobius niger*	<0.1 / ND	ND / ND	ND / ND	ND / ND	ND / ND
Tench *Tinca tinca*	ND / ND	ND / ND	ND / ND	ND / ND	ND / ND
Three-spined stickleback *G*. *aculeatus*	ND / <0.1	ND / ND	ND / ND	ND / ND	ND / ND
Cyprinids Cyprinidae	5.5 / ND	31.1 / ND	ND / ND	10.6 / ND	33.8 / ND
Whitefishes *Coregonus* spp	ND / 18.8	ND / 9.1	1.4 / 15.6	0.1 / 15.4	ND / 4.5
Percids Percidae	ND / ND	2.1 / ND	10.7 / ND	ND / ND	3.7 / ND
European flounder or plaice *Plat*. *flesus* or *Pleur*. *platessa*	ND / 7.5	ND / ND	ND / ND	ND / ND	ND / ND
Sculpins Cottidae	<0.1 / 0.1	ND / 1.7	ND / ND	ND / 10.7	ND / ND
Clupeids Clupeidae	5.9 / ND	ND / ND	ND / ND	0.1 / ND	ND / ND
Daces *Leuciscus* sp.	ND / ND	ND / ND	ND / 6.3	ND / ND	ND / ND
Gobies Gobiidae	<0.1 / ND	<0.1 / ND	ND / ND	0.2 / ND	ND / ND

### Prey fish fatty acids and stable isotopes for long-term diet assessment

**Fig 2:**
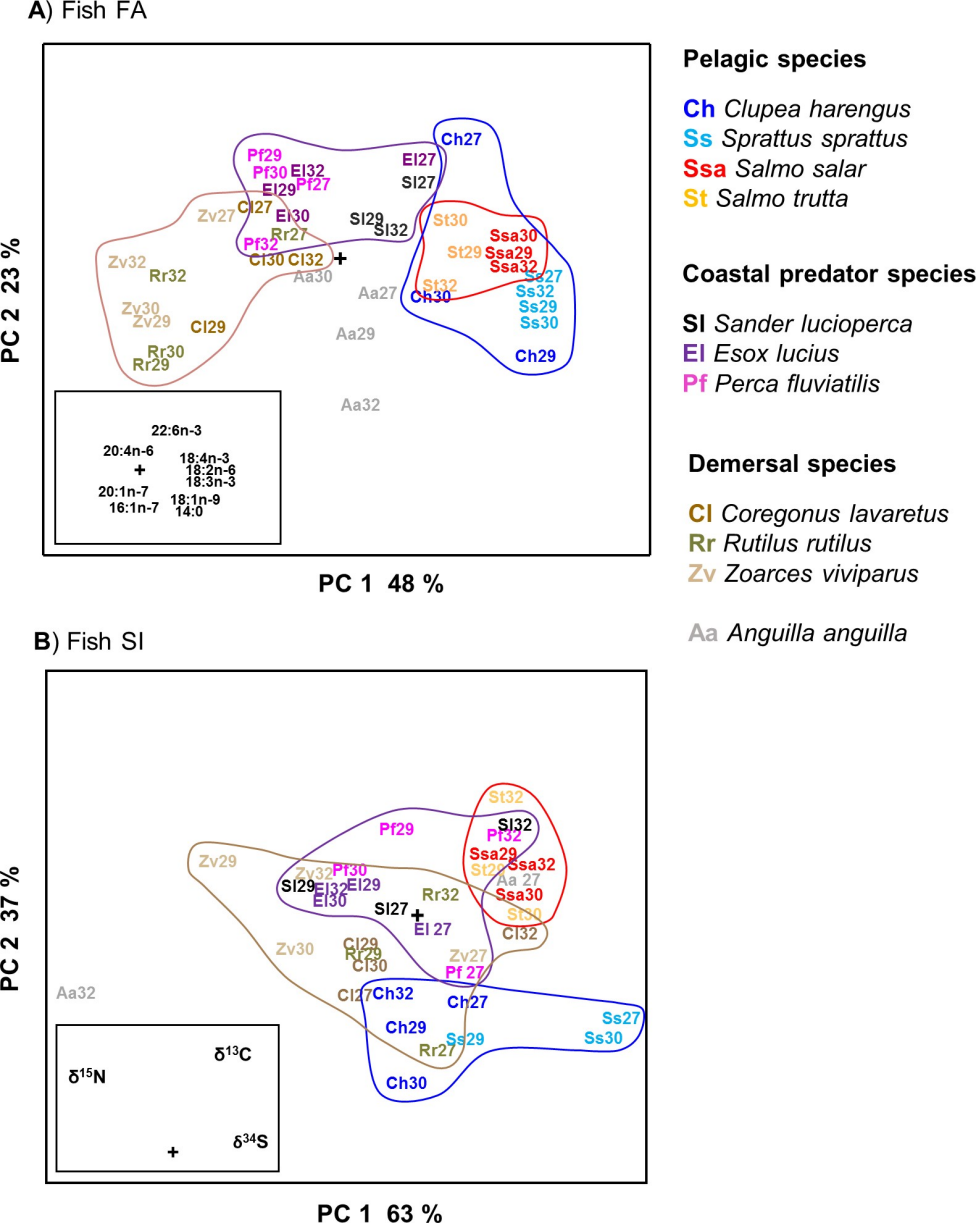
PCA scores plots of A) FA and B) SI means for the 11 key prey fish species from ICES-subdivisions 27, 29, 30 and 32. The species and their habitat classification are shown on the right. Target sample number per species for each area was 6 specimens (Table A in S1 Table). Loadings plots of the variables were added as inserts. Paired SIMCA tests (*P* < 0.05) of the statistical significance of the compositional differences between and within species are presented in supporting information (Tables B-C and Text B in S1 Table).

The FAs and SIs of 11 key prey species representing pelagic, coastal or demersal habitats were analysed by PCA and the mean compositions in each area were plotted for each species (Fig 2). The first principal components (PC 1) explained 48 and 63% of the total data variation of the fish FA and SI profiles, respectively, and represented a shift from demersal to pelagic species where the FAs grouped the species according to their ecology more clearly than the SIs. Pelagic species (herring, sprat, Atlantic salmon and sea trout) were characterized by their high relative contents of 18:4n-3, 18:2n-6 and 18:3n-3, whereas demersal species (eelpout, roach and common whitefish) contained higher relative amounts of 20:4n-6, 20:1n-7 and 16:1n-7. Coastal predators (pikeperch, pike and perch) showed intermediate composition with a slight enrichment of 22:6n-3. European eel was characterized by high contents of monounsaturated FAs and 14:0. Despite the poorer general separation when using SIs, the second axis (PC 2) separated pelagic plankton feeding herring and sprat from the pelagic predators, Atlantic salmon and sea trout, a separation not found when using FAs as variables. Coastal predators showed high values for both δ^15^N and δ^13^C.

Results from inter- and intraspecies comparisons by PCA and SIMCA using the FA and SI data (and the original SI values) in each ICES area are presented as supporting information (Tables A-D and Texts A-B in S1 Table). According to SIMCA, 65% of interspecies comparisons of fish tissue FAs within and between ICES areas reached statistical significance, while the corresponding number for SI comparisons was 45%. SIMCA revealed statistically significant intraspecies differences in FA composition between all ICES areas for herring, Atlantic salmon, perch, common whitefish and eelpout. In the case of SIs, SIMCA revealed statistically significant intra-specific differences only in sprat (SD27 vs 29 significant), pikeperch (SD27 vs 29, SD29 vs 32), pike (SD29 vs 32, SD30 vs 32) and eelpout (SD29 vs 30, SD30 vs 32).

### Indicators of mid- and long-term diet

**Fig 3:**
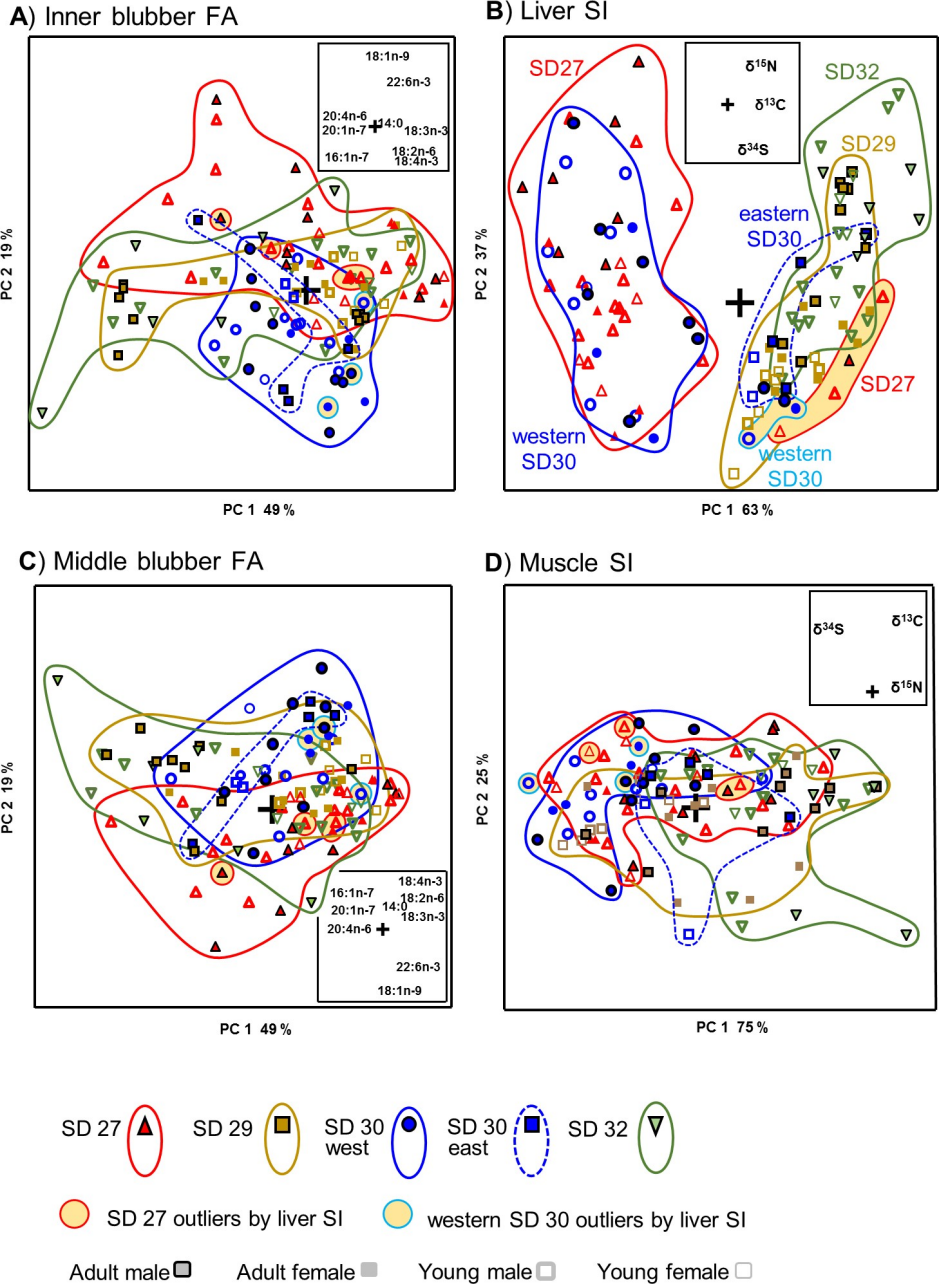
PCA scores plots of the mid-term markers A) inner blubber FA, B) liver SI, and the long-term markers C) middle blubber FA and D) muscle SI data for grey seal individuals (n = 108) collected from ICES-subdivisions 27, 29, 30 and 32. Symbol key is presented below figures. Loadings plots of the variables were added as inserts. Without any subgrouping of the individuals, according to age or sex, the paired SIMCA tests (*P* < 0.05) showed no significances in any comparisons between ICES areas.

At first, the data of all grey seal individuals (n = 108) studied for tissue chemical markers were subjected to PCA and possible differences were examined in their inner blubber FAs (Fig 3A), liver SIs (Fig 3B), middle blubber FAs (Fig 3C) and muscle SIs (Fig 3D). The FAs in the inner and middle blubber did not group the specimens according to area, gender or age (Fig 3A and 3C). However, liver SIs clearly segregated seals from SD27 and western SD30 from another group containing all seals from SD29, SD32 and eastern SD30 (Fig 3B). However, this latter group also contained 4 individuals from SD27 and 3 individuals from western SD30, all captured in Aug-Nov (these subgroups were indicated by yellow background in Fig 3B, and their individuals were also highlighted in Fig 3A, 3C and 3D, although they did not form groups when plotted according to these other markers). Thus, the seals from the Swedish coast included 7 individuals with Finnish coast SI signatures whereas all the individuals collected off the Finnish coast had similar SIs. The seals from Finland had higher values for δ^15^N, δ^13^C and δ^34^S, and the 7 mentioned individuals caught on the Swedish side showed SIs characteristic for the individuals from Finland. In contrast, the muscle SIs did not distinguish any groups (Fig 3D). If the sample groups of each area were compared as such, without taking into consideration possible exceptional migrating individuals and without any subgrouping of the individuals according to age or sex, the paired SIMCA tests (*P* < 0.05) showed no significances in any comparisons between ICES areas. However, if an age group is studied separately, statistically significant separations can be found. When the subadult males collected from bottom fykes and shot in open water area (for these collections several individuals were available from all SD areas), the individuals grouped according to ICES area (Fig A in S1 Fig). Within the subadult males the inner blubber FA and liver SI profiles were significantly different between the individuals collected from certain Baltic SDs (paired comparisons by SIMCA *P* < 0.05: inner blubber 27/30 and 29/32; liver SI 29/30 and 30/32; middle blubber FA and muscle SI not significant). The adult males are addressed below but the low sample numbers prevented similar comparisons with the females.

To compare the separation power of the FAs and SIs, data from seals with detailed information about sex, age, area and type of fishing gear (defines the recent feeding habitat) were subjected to PCA. Such strictly defined groups could only be formed of adult males from the Finnish coast caught from trawls or different types of fykes (T, S and B, total n = 11). Thus, three groups were formed from these males that were from the same area and had been caught there with the same type of gear (Table 5).

**Table 5:** The accurate background information on 11 adult male grey seal individuals that were grouped according to the catching gear type and used to test the ability of the tissue chemical markers to indicate differences in feeding area or prey type.

Group	ID	Age (yrs)	Blubber depth (mm)	ICES area	Bycaught in	Target fish species
T	1588	16	33	30	trawl	herring
T	1613	28	42	30	trawl	herring
T	1610	10	60	30	trawl	herring
T	1629	19	45	30	trawl	herring
S	1606	7	48	32	surface fyke	salmonids, common whitefish
S	1593	10	30	32	surface fyke	salmonids, common whitefish
S	1553	15	21	32	surface fyke	salmonids
S	1526	15	27	32	surface fyke	salmonids
B	1574	17	36	32	bottom fyke	perch, pikeperch, cyprinids
B	1598	11	42	29/32*	bottom fyke	pikeperch
B	1624	10	42	32	bottom fyke	perch, pikeperch, cyprinids

Individual identity code (ID), age, blubber depth on sternum, ICES-area and the gear type (T = trawl, S = surface fyke, B = bottom fyke) where the seal individual was collected from, and the fish species targeted with the gear are presented.

* Individual 1598 was caught at the border of SD29 and 32.

**Fig 4:**
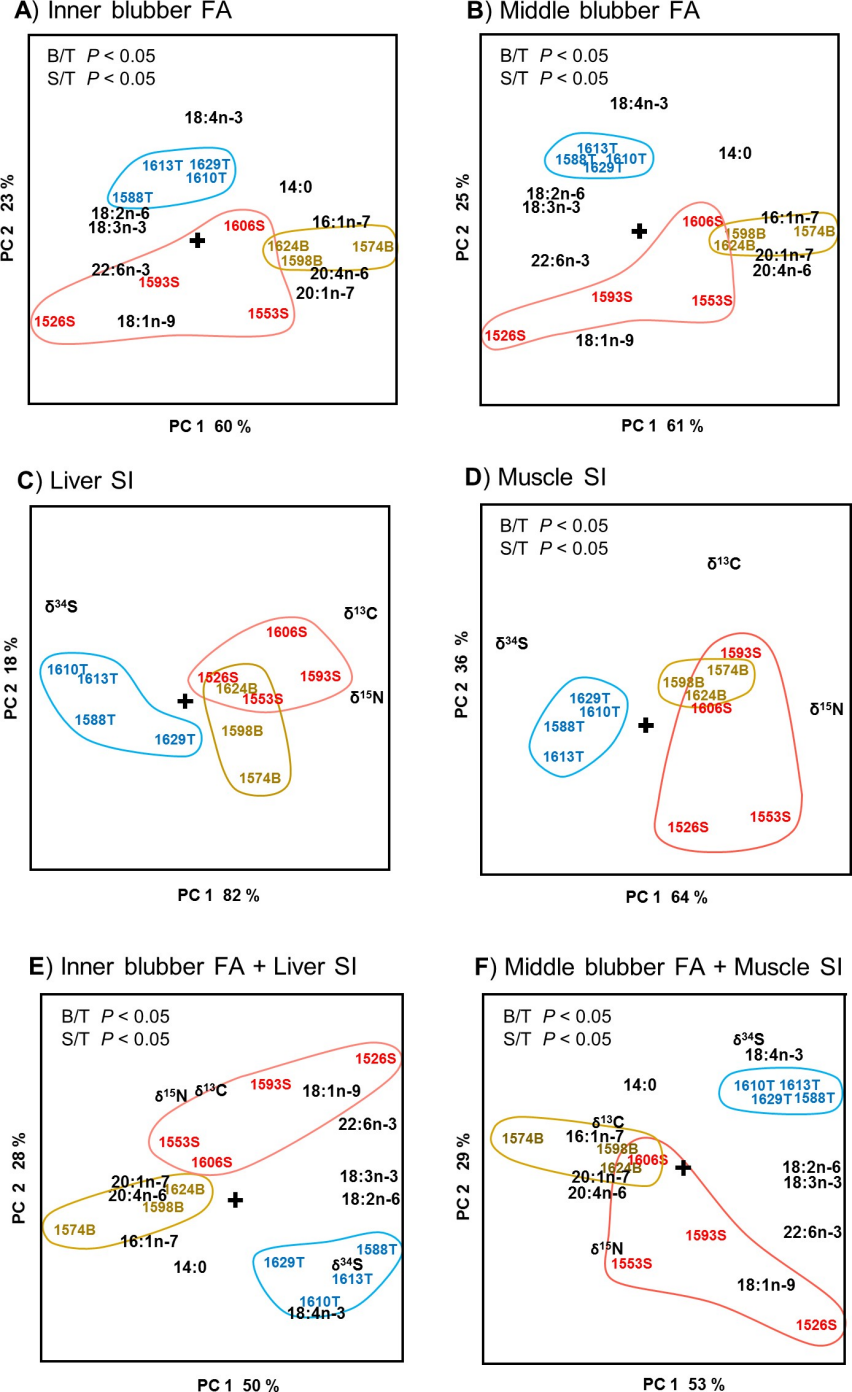
PCA biplots of A) inner blubber FA, B) middle blubber FA, C) liver SI, D) muscle SI, E) combined inner blubber FA and liver SI data, and F) combined middle blubber FA and muscle SI data for 11 Finnish adult male grey seal individuals. Results of paired SIMCA tests of the statistical significance of the compositional differences (*P <* 0.05) are listed in the upper-left corner of the plots (no in panel C). T = bycaught in trawl, S = bycaught in surface fyke, B = bycaught in bottom fyke. On the plot, the numbers combined with the gear specification letter indicate individual seal identification code.

The segregation of these groups of adult males were examined by using inner blubber FAs (Fig 4A) and liver SIs (Fig 4C), representing the mid-term diet, and middle blubber FAs (Fig 4B) and muscle SIs (Fig 4D), representing the long-term diet. In addition, to study whether combining the FA and SI markers could improve segregation power, the PCA was repeated by using combined data of the mid-term indicators (Fig 4E) as well as the long-term indicators (Fig 4F). Several of these comparisons revealed statistically significant differences between the samples from a certain catch area and trap type. Individuals of SD30/trawl group (T) significantly separated from those in SD32/surface fyke (S) and SD32/bottom (B) groups when using either FA or FA+SI data as loadings. The trawl group contained higher percentages of pelagic C18 polyunsaturated FAs (PUFAs), especially 18:4n-3. At the same time these individuals had low percentages of 16:1n-7, 20:1n-7 and 20:4n-6, plentiful in demersal fish. However, the SIs alone showed significant difference only between the trawl (T, with high δ^34^S) versus coastal fyke groups (S and B, with high δ^13^C and δ^15^N) when using muscle SIs as variables. When using Liver SIs, PCA followed by SIMCA showed no statistically significant differences. The individuals that were bycaught in the pelagic surface or coastal bottom fykes (S vs B) did not differ from each other in any of the comparisons by FAs and SIs, alone or combined. Further comparisons on the separation power of FAs and SIs using a larger set of adult male individuals are presented in Fig B in S1 Fig.

For consistency, all the multivariate comparisons were performed by using metric PCA and SIMCA. To ensure that the results of the comparisons using the limited data of 11 adult males were not biased, these PCA and SIMCA comparisons (Fig 4) were repeated by non-metric nMDS and ANOSIM (S2 Fig). The results were essentially the same, the nMDS/ANOSIM confirming the statistically significant separations indicated by PCA/SIMCA. The only marked difference was that the non-metric approach detected a significant difference in liver SI profiles of the adult males collected from surface fyke and trawl (S vs T), which according to the metric analysis was not significant.

## Discussion

In the current study, the power of different methods to determine marine mammal diet or feeding ecology was compared. Studies using several complementary methods, allowing to confirm the results of one dietary proxy with another, have not been conducted on any seal species in the Baltic Sea previously, and there are no studies applying all the proxies included in this work from any other marine mammal population either. Hence, in the absence of reference studies, our study is unique. The first published surveys of Baltic grey seal diet used gut prey remains and were based on material from the 1960-70s [58,59]. Lundström et al. [8,29] and Kauhala et al. [9] used material from the early 2000s. Thereafter, Baltic grey seal diet has not been studied in a decade. However, the large spatial and temporal variability of Baltic food webs and fish stocks [60–63] makes this grey seal population an excellent model of studying the power of different diet monitoring methods. Parallel to the ecosystem and food web change the grey seal population is growing and may adopt new foraging habits.

### Short-term methods address dietary fish species

Morphological identification of undigested prey remains relies on expertise and reference collections but enables estimates of ingested prey sizes and biomass. DNA metabarcoding of gut contents provides more exact prey identification but does not provide information about prey size, and experience in converting DNA sequence proportions to biomass does not yet exist. Thus, the estimated mass proportions from the HP analyses and the DNA sequence proportions are not fully comparable to each other. Despite these limitations, the DNA metabarcoding clearly demonstrated prey taxa that are underestimated by the HP analyses.

In accordance with the study conducted by Lundström et al. [8], herring was in general the most frequently consumed prey. The digestive tract material of Lundström and co-workers was collected during 2001–2005, all seasons and seal age classes included, and it covered an area from the Gulf of Bothnia till the southern Baltic, but without SD32, included in the current study. According to the short-term methods applied in this work, other important prey were perch, eelpout, cyprinids, common whitefish, sprat and pikeperch, however with marked differences between areas. Compared to the morphological study of Lundström et al. [8], the current study gave higher dietary shares for perch, pikeperch and cyprinids likely explained by the inclusion of samples from SD32, Gulf of Finland in this study (Tables 3 and 4). When comparing the morphological HP and DNA data, *Salmo* species were found to be underrepresented by the HP analysis in male seals (Tables 1,3 and 4).

The short-term methods suggested dietary differences between age groups and areas, also found in the study by Lundström et al. [8]. Since these methods are limited in giving snapshot estimates on the diet, no firm conclusions could be made on the dietary differences between males and females due to the low number of female samples. The differences in diet between areas describe a shift from herring dominance in the central parts to percid and cyprinid (especially bream) dominance in the Gulf of Finland, and importance of perch in the western Baltic Proper. The prey of the Baltic grey seals is distinguished from the diets of grey seals of the Atlantic, where herring is a minor dietary component but gadoids, flatfish and sandeels are frequently consumed [64,65].

### Chemical tissue markers require reference prey library

Regardless of accurate prey identification, no short-term method reveals the integrated average prey of a free-ranging marine mammal, which may migrate and thus at different times exploit different habitats and prey. Attempting to attain data of temporal representativeness from short-term gut samples would require frequently repeated hunt. Diet assessment using FAs and SIs offer long-term dietary estimates but with the drawback of failing to reach firm prey species identification. Baltic grey seals have so far been studied little for tissue FAs [66,67], and tissue SIs have previously only been studied for Baltic ringed seals (*Phoca hispida bothnica*) [68].

Successful food web studies require a representative reference library of prey FAs and SIs and that the prey species have characteristically different chemical markers. Used together, FAs and SIs are complementary since they are proxies of different dietary components. While the FAs are derived from dietary lipids, the δ^15^N values and in this work also the δ^13^C values, analysed by using delipidated samples [69], represent proteins. FA profiling has previously proven to be an effective method to study predator foraging ecology and may even indicate specific prey species [17,18] but the power of the method in such species-level diet determination has also been questioned [70]. The habitat-specific FA profiles of fish have their origin in plankton or microorganisms mediated by invertebrates to the tissues of the fish [71,72]. The pelagic fish species herring, sprat, Atlantic salmon and sea trout obtained especially high proportions of C18 PUFAs, the 18:4n-3 being especially abundant in herring and sprat. The demersal species were distinguished by the FAs 16:1n-7, 20:1n-7 and 20:4n-6.

Comparisons of SIs of prey and predator provides at least information on the trophic levels of the dietary fish and the area they originate from, but mixing models can also identify between limited number of established principal prey taxa [73,74]. In general, marine biomes tend to have high δ^15^N and δ^13^C but high δ^34^S may come from freshwater input, sediments, precipitations and various anthropogenic sources and thus may offer information on foraging area [46]. Among the Baltic fish, the δ^34^S was enriched in all studied *Salmo* species. To our knowledge, the δ^34^S has not been used in the studies of Baltic food webs before but should become a frequently analyzed element since aquatic primary producers have been reported to have large variation of δ^34^S values, and their level of trophic‐step fractionation is low, which makes the element useful in revealing food web relations [75,76]. The early years the *Salmo* species spent in river or their estuarine dietary sources, with sulphur supply different from the pelagic sea areas, may have influence on this value (Table D in S1 Table).

Although the SI ratios in general had weaker power than the FAs in grouping the studied fish species according to their habitat, δ^13^C managed to subgroup pelagic species (all high in δ^34^S) by separating the predators salmon and trout (high δ^13^C, high δ^34^S) from the planktivorous herring and sprat (low δ^13^C, high δ^34^S, Fig 2). This further separation likely stems from the δ^13^C value increasing towards higher trophic levels. According to literature, pelagic species in general have higher δ^13^C values than coastal species, which are affected by freshwater run-off with soil organic matter having low δ^13^C values [46]. In line with this, the Atlantic salmons analyzed for this study were caught in the Baltic Proper. The Baltic demersal fishes were slightly enriched in δ^15^N likely due to the fact that the trophic level of sediment biota is higher than that of pelagic plankton [77].

### Mid- and long-term methods reveal individual specialization for area and type of prey

Provided that no prior subgrouping of the seal individuals was made to diminish biological variation, the only chemical tissue marker that grouped the seals according to the studied parameters (ICES area, sex, age or cause of death (bycaught/hunted)) was the liver SIs (with the time window of weeks and reflecting dietary protein component), which indicated that the individuals from SD27 and western SD30 had similar signatures differing from those in the SD29 and SD32 samples. The SD29 seals were all from the area between Åland Islands and Turku. This west-east pattern was broken by 7 seals (6.5%) caught on the west coast of SD27 and SD30 but with liver SI signatures similar to most of the individuals caught in the east, which suggests westward migration. These possibly migrated individuals were of different age and sex, and had blubber FA compositions similar to those of the other SD27 and SD30 western individuals. This leaves similar foraging area in the past, better indicated by SIs than FAs [46,75], as a tempting unifying factor. Since this finding is purely chemical marker data-driven, we unfortunately have no direct telemetric or other proof that these individuals would have been migrated. In theory, specific locations with element SI characteristics different from the surroundings may exist. However, the proportion of migrating seals suggested by this study is in the same range with a recent GPS tracking study of male grey seal of the Baltic, reporting that 12% of the individuals migrated (moved out from a 60x120 km^2^ area) during a couple of months’ time [16].

In the case of blubber FAs, the lack of distinct sample groups in the PCA of all 108 individuals made it difficult to recognize the dietary origin of the variation in blubber FA profiles. However, reducing biological variation among the individuals studied may help in relating tissue FA profile differences to feeding area and diet, and indeed among the subadult males the inner blubber FA and liver SI profiles grouped individuals according to the SD area they were collected in (Fig A in S1 Fig). When studying free-ranging wild specimens, ideally, the influence of diet on the chemical markers can be studied by comparing the markers between groups of individuals of the same gender and age group, and collected in the same area and from same type of fishing gear located in similar habitats. In this study, such groups of adult males, presumably having similar foraging ecology, had similar tissue chemical markers and were successfully grouped by metric PCA/SIMCA and non-metric nMDS/ANOSIM. Despite the sample was small, this suggests individual dietary specialization not detectable if the studied individuals had had opportunistic foraging habits. Thus, the dietary effect on this marker was not questionable. In addition, the blubber FA profiles of the males from the area/gear subgroups were enriched by the FAs characteristic for the fish usually caught in that area by that specific gear type. This dietary effect was sustained also when using a larger dataset (Fig B in S1 Fig). Recent studies on grey seal males’ foraging behaviour in the Baltic have shown clear site fidelity to the same area with about 100 km range [16]. In SD30, Königson et al. [15] detected specialization of adult grey seal males to salmon catching surface fykes. In addition, based on bycatch statistics of the present data, Kauhala et al. [5] classified males from all age groups as potential “problem seals”. Considering all the dietary proxies employed in this work, different commercially exploited fish species, *e*.*g*. herring, are consumed by both young and adult seal individuals. The tissue chemical markers, however, revealed individual long-term specialization for a certain habitat and type of prey for the adult males, and the DNA analyses, especially, confirmed that Atlantic salmon was included in their diet. These findings imply that the adult males are the most likely individuals to cause local and sustained loss of catch of the most valuable fish, and gear damage.

Available information on the rates of absorption and turnover of FAs and SIs in seal tissues [20,21,75] suggest that the FA and SI values of the inner blubber and liver reflect the diet consumed 2–3 weeks prior to sampling, and the chemical signatures of the middle blubber and muscle tissue should represent diet assimilated up to 2–3 months prior to sampling. Hence, we prefer the naming “mid-term” diet when inner blubber FAs and liver SIs are used in diet assessment, while “long-term” diet would be described by middle blubber FAs and muscle SIs. Comparison of these marker profiles of the adult males from the well-defined subgroups revealed that the best separation power in these week‒month time scales is obtained with FAs. Combined use of FA and SI data as loadings did not improve the separation power. The FAs and SI ratios of the adult male grey seals complied with the characteristic FAs and SI ratios found in the key fish of their catching habitat. For example, PCA showed a surprisingly strong correlation between the high liver δ^34^S and blubber 18:4n-3 in the adult male seals bycaught in trawls. Thus, by this first use of the δ^34^S to study foraging ecology of the seals we could define δ^34^S as a marker of consuming SD30 pelagic fish. The fact that both mid-term and long-term markers separated the seal individuals in similar ways suggests individual long-term specialization for a certain type of prey and habitat.

## Conclusions

Analysis of gut contents was required to identify prey species, and both morphological analysis of prey hard parts and DNA metabarcoding showed clear dietary differences between age groups and areas. Concerning the foraging ecology of the seals, these proxies of the very recent diet cannot reveal potential specialization of individuals for certain feeding area or prey type. For this purpose the mid- and long-term markers can be used. The bycaught adult males formed distinctive groups having similar FA and SI markers, which resembled the marker patterns of the fish caught in the area by the gear type in which the seals were found (Fig 4, S2 Fig). A probable interpretation was that these adult males had been using the same foraging areas for long, and perhaps raiding the gears there repeatedly. Since these groupings by the mid- and long-term dietary markers were the most obvious in the adult males, this is likely a consequence of specialized foraging or male territorial behaviour. Selective removal of problem seals has been suggested to mitigate the conflicts between seals and coastal fisheries. In the light of the current study, if implemented, such selective culling should be directed towards the adult males that were found to be the most specialized in their foraging tactics, and may locally cause significant economic losses for fisheries in the form of gear damage and loss of catch. Differences in mid-term diet, reflecting foraging areas, were also seen in the liver SIs, which also may have distinguished a few migrated seals.

This study suggests a combination of multiple diet estimation methods as the optimal protocol to assess as detailed information as possible about feeding habits of aquatic top predators. Efficient use of the dietary methods, however, sets high requirements for recording detailed background information on the studied individuals, which is a prerequisite for discovering dietary subgroups in large diverse data sets.

## Supporting information

S1 Table Fish fatty acid (FA) and stable isotope (SI) sample numbers, SIMCA results of fish FA and SI, and fish SI original values. (PDF)

S1 Fig PCA and SIMCA of tissue FAs and SIs using data subsets of subadult and adult male grey seals. (PDF)

S2 Fig MDS and ANOSIM analyses of the tissue chemical markers in 11 adult males with accurate background information. (PDF)

S1 Data Original fish and seal data. (XLSX)
