# Supplementary material for: Complementary methods assessing short and long-term prey of a marine top predator ‒ Application to the grey seal-fishery conflict in the Baltic Sea
Source: PLoS One. 2019 Jan 2;14(1):e0208694. doi: 10.1371/journal.pone.0208694 (PMC6314633; doi:10.1371/journal.pone.0208694)
Supplement: S1 Table — (PDF) [file pone.0208694.s001.pdf]

S1 Table. Fish fatty acid (FA) and stable isotope (SI) sample numbers, SIMCA results of fish FA and SI, and fish SI original values

Table A. Fatty acid (FA) and stable isotope (SI) sample numbers, n(FA) and n(SI), for the 11 most important fish prey of the Baltic grey seals arranged according to the ICES-defined sea subdivisions (27, 29, 30 and 32).

**Prey items**	**n(FA) / n(SI)**				
	**27**	**29**	**30**	**32**	**Total**
Clupea harengus	6/6	6/5	6/6	0/6	18/23
Sprattus sprattus	6/6	6/5	6/6	6/0	24/17
Salmo salar	0/0	3/6	6/6	6/6	15/18
Salmo trutta	0/0	6/5	4/3	6/6	16/14
Sander lucioperca	6/6	6/6	0/0	6/6	18/18
Esox lucius	5/5	6/6	6/6	6/6	23/23
Perca fluviatilis	6/6	6/6	6/6	6/6	24/24
Coregonus lavaretus	6/6	6/6	6/6	6/6	24/24
Rutilus rutilus	6/6	6/6	6/0	6/6	24/18
Zoarces viviparus	6/6	6/7	6/6	6/6	24/25
Anguilla anguilla	6/6	6/0	5/0	6/6	23/12
All species					233/216

Text A. Interspecies comparisons (Tables B-D)

According to SIMCA, 65% of interspecies comparisons of fish tissue mean FA values within and between ICES areas reached statistical significance, while the corresponding number for mean SI value comparisons was 45%.

Table B. Interspecies SIMCA-comparisons of fish FAs from ICES-areas 27, 29, 30 and 32. The SD area marked in a cell indicates significant (P <0.05) difference in the fatty acid composition between the two species in that area.

	Ch	*Ss*	*Ssa*	*St*	*Sl*	*El*	*Pf*	*Cl*	*Rr*	*Zv*	*Aa*
Ch		27, 30	30	30			30	30	30	27	30
Ss	27, 30		30, 32	30, 32			30, 32	30	30	32	30, 32
Ssa	30	30, 32		30, 32			30, 32	30	30	32	30, 32
St	30	30, 32	30, 32				29, 30, 32	29, 30	29, 30	29, 32	29, 30, 32
Sl											
El											
Pf	30	30, 32	30, 32	29, 30, 32				29, 30	29, 30	29, 32	29, 30, 32
Cl	30		30	29			29, 30		29, 30	29	29, 30
Rr	30	30	30	29, 30			29, 30	29, 30		29	29, 30
Zv	27	27, 32	32	29, 30			29, 30	29	29		29, 32
Aa	30	30, 32	30, 32	29, 30, 32			29, 30, 32	29, 30	29, 30	29, 32	

Ch = Clupea harengus, Ss = Sprattus sprattus, Ssa = Salmo salar, St = Salmo trutta, Sl = Sander lucioperca, El = Esox lucius, Pf = Perca fluviatilis, Cl = Coregonus lavaretus, Rr = Rutilus rutilus, Zv = Zoarces viviparus, Aa = Anguilla anguilla.

Table C. Interspecies SIMCA-comparisons of fish SIs from ICES-areas 27, 29, 30 and 32. The SD area marked in a cell indicates significant (P <0.05) difference in the stable isotope value profile between the two species in that area.

	Ch	*Ss*	*Ssa*	*St*	*Sl*	*El*	*Pf*	*Cl*	*Rr*	*Zv*	*Aa*
Ch			29, 30, 32	29, 30, 32	32	29, 30, 32				29, 30, 32	
Ss			29	29	27, 29	27, 29	29, 30			29	27
Ssa	29, 30, 32	29				30, 32				29, 32	
St	29, 30, 32	29				30, 32	30	29	29	29, 30, 32	
Sl	29, 32	27, 29				32				32	
El	29, 30, 32	27, 29	30, 32	30, 32	32			30		29, 30	
Pf		29, 30		30					29		
Cl				29		30					
Rr				29			29			29	
Zv	29, 30, 32	29	29, 32	29, 30, 32	32	29, 30			29		
Aa		27									

Ch = Clupea harengus, Ss = Sprattus sprattus, Ssa = Salmo salar, St = Salmo trutta, Sl = Sander lucioperca, El = Esox lucius, Pf = Perca fluviatilis, Cl = Coregonus lavaretus, Rr = Rutilus rutilus, Zv = Zoarces viviparus, Aa = Anguilla anguilla.

Table D. **Mean isotopic values for δ^{13}C, δ^{15}N and δ^{34}S in fish tissues from ICES areas 27, 29, 30 and 32.** All mean values are given in ‰.

**SD27**	***Ch***	***Ss***	***Ssa***	***St***	***Sl***	***El***	***Pf***	***Cl***	***Rr***	***Zv***	***Aa***
**δ^{34}S**	19.45	21.84			19.70	18.52	18.09	16.16	15.51	18.97	18.42
**δ^{15}N**	11.74	9.15			14.95	12.93	11.34	12.10	9.34	12.03	11.72
**δ^{13}C**	-21.30	-19.64			-21.76	-19.92	-19.39	-20.23	-18.20	-19.93	-17.04
**SD29**	***Ch***	***Ss***	***Ssa***	***St***	***Sl***	***El***	***Pf***	***Cl***	***Rr***	***Zv***	***Aa***
**δ^{34}S**	16.17	16.96	20.41	21.25	15.87	17.68	17.04	15.05	15.86	13.40	
**δ^{15}N**	10.69	9.80	13.70	14.08	14.35	14.75	13.97	11.78	12.28	14.86	
**δ^{13}C**	-19.54	-19.14	-19.24	-20.26	-19.32	-20.20	-17.60	-18.05	-19.09	-18.41	
**SD30**	***Ch***	***Ss***	***Ssa***	***St***	***Sl***	***El***	***Pf***	***Cl***	***Rr***	***Zv***	***Aa***
**δ^{34}S**	16.86	21.46	21.03	21.38		17.66	16.40	16.15		15.07	
**δ^{15}N**	10.40	9.40	13.12	12.77		15.35	14.17	12.32		13.35	
**δ^{13}C**	-21.23	-20.51	-19.81	-20.20		-.21.20	-18.86	-19.25		-19.85	
**SD32**	***Ch***	***Ss***	***Ssa***	***St***	***Sl***	***El***	***Pf***	***Cl***	***Rr***	***Zv***	***Aa***
**δ^{34}S**	16.19		21.81	21.73	21.10	15.32	19.94	19.45	17.47	15.37	11.27
**δ^{15}N**	11.44		13.73	15.13	13.96	13.37	13.44	11.46	12.45	14.07	15.07
**δ^{13}C**	-19.42		-19.71	-18.94	-18.79	-18.23	-18.03	-18.80	-18.07	-18.53	-21.74

Ch = Clupea harengus, Ss = Sprattus sprattus, Ssa = Salmo salar, St = Salmo trutta, Sl = Sander lucioperca, El = Esox lucius, Pf = Perca fluviatilis, Cl = Coregonus lavaretus, Rr = Rutilus rutilus, Zv = Zoarces viviparus, Aa = Anguilla anguilla.

Text B. Intraspecies comparisons

SIMCA revealed statistically significant intraspecies differences in FA composition between all ICES areas for herring, Atlantic salmon, perch, common whitefish and eelpout. In the case of SIs, SIMCA revealed statistically significant intraspecies differences only in sprat (SD27 vs 29 significant), pikeperch (SD27 vs 29, SD29 vs 32), pike (SD29 vs 32, SD30 vs 32) and eelpout (SD29 vs 30, SD30 vs 32).
