# Supplementary material for: Complementary methods assessing short and long-term prey of a marine top predator ‒ Application to the grey seal-fishery conflict in the Baltic Sea
Source: PLoS One. 2019 Jan 2;14(1):e0208694. doi: 10.1371/journal.pone.0208694 (PMC6314633; doi:10.1371/journal.pone.0208694)
Supplement: S1 Fig — (PDF) [file pone.0208694.s002.pdf]

S1 Fig. PCA and SIMCA of tissue FAs and SIs using data subsets of subadult and adult male grey seals

When subadult males collected from bottom fyke nets (B) or hunted in open water area (O), which we were able to collect in all the SD areas, were subjected to PCA, the samples from SD27 versus SD30 and SD29 versus SD32 differed in terms of inner blubber FA composition (Fig A, panel a). The liver SIs showed spatial groupings and significant difference between SD29 and SD30, and between SD30 and 32 (Fig A, panel b). The small number of female samples (subadult n = 15, adults n = 16) from diverse areas did not allow reliable comparative analysis.

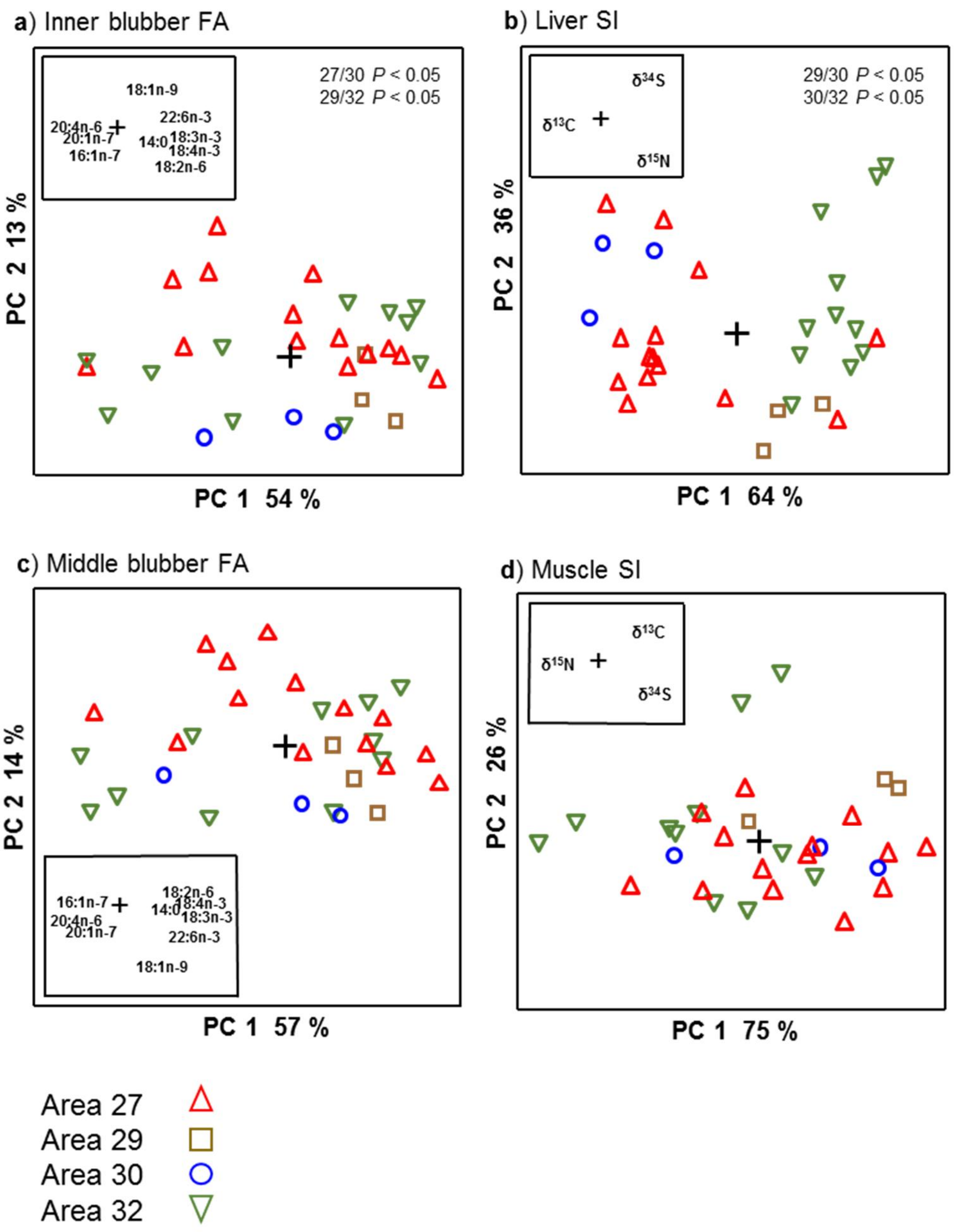

Fig A. PCA scores plots of **a**) inner blubber fatty acid (FA), **b**) liver stable isotope (SI), **c**) middle blubber FA and **d**) muscle SI data for subadult Finnish and Swedish male seal individuals (n = 31) from individuals caught by bottom fyke (B) and hunted in open water area (O) from ICES-areas 27, 29, 30 and 32. Loadings plot of the variables were added as inserts. SIMCA comparisons ($P < 0.05$) were applied to two areas at a time, and statistical significances, when found, were presented in upper-right corner of the plot.

To examine the separation power of FAs and SIs in the data of the 11 bycaught adult males (see Fig 4 of the main article) but now with hunted males (O = open-water area, away from fishing gear, C = close to gear and F = at fish farm), PCA was applied to these combined data of 21 adult male seals collected from Finnish waters (Fig B). Adding more adult males with various background did not affect the grouping of the original 11 bycaught individuals collected from specific areas and gear types. Paired SIMCA tests of the statistical significance of the compositional differences ($P < 0.05$) were inserted in the upper-left corner of the plots.

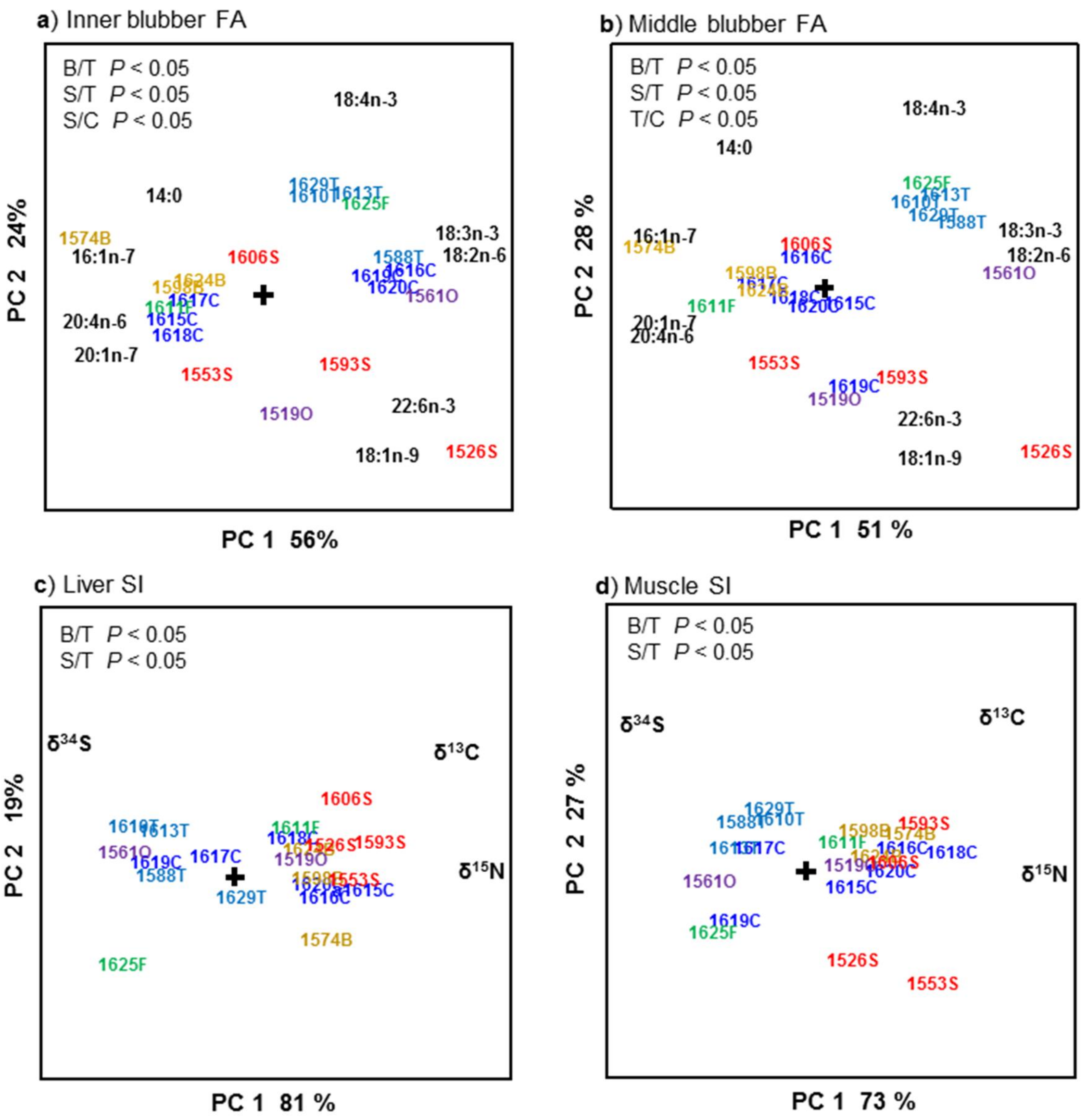

Fig B. PCA scores plots of **a**) inner blubber FA, **b**) liver SI, **c**) middle blubber FA and **d**) muscle SI for Finnish adult male seal individuals (n = 21) from groups T, S, B, O, C and F. Paired SIMCA tests of the statistical significance ($P < 0.05$) of the compositional differences presented in upper-left corner of the plots. T = trawl, S = surface fyke, B = bottom fyke, O = open water, C = close to fishing gear, F = by fish farm. Numbers preceding gear specification indicate individual seal identification code.
