# Supplementary material for: Complementary methods assessing short and long-term prey of a marine top predator ‒ Application to the grey seal-fishery conflict in the Baltic Sea
Source: PLoS One. 2019 Jan 2;14(1):e0208694. doi: 10.1371/journal.pone.0208694 (PMC6314633; doi:10.1371/journal.pone.0208694)
Supplement: S2 Fig — (PDF) [file pone.0208694.s003.pdf]

S2 Fig. MDS and ANOSIM analyses of the tissue chemical markers in 11 adult males with accurate background information

The PCA and SIMCA analyses (Fig. 4 of main text) using the limited data of 11 adult males (with accurate background information; Table 5 of the main text) were repeated by non-metric MDS and ANOSIM (Fig). The results revealed to be essentially the same, the MDS/ANOSIM confirming the statistically significant separations indicated by PCA/SIMCA.

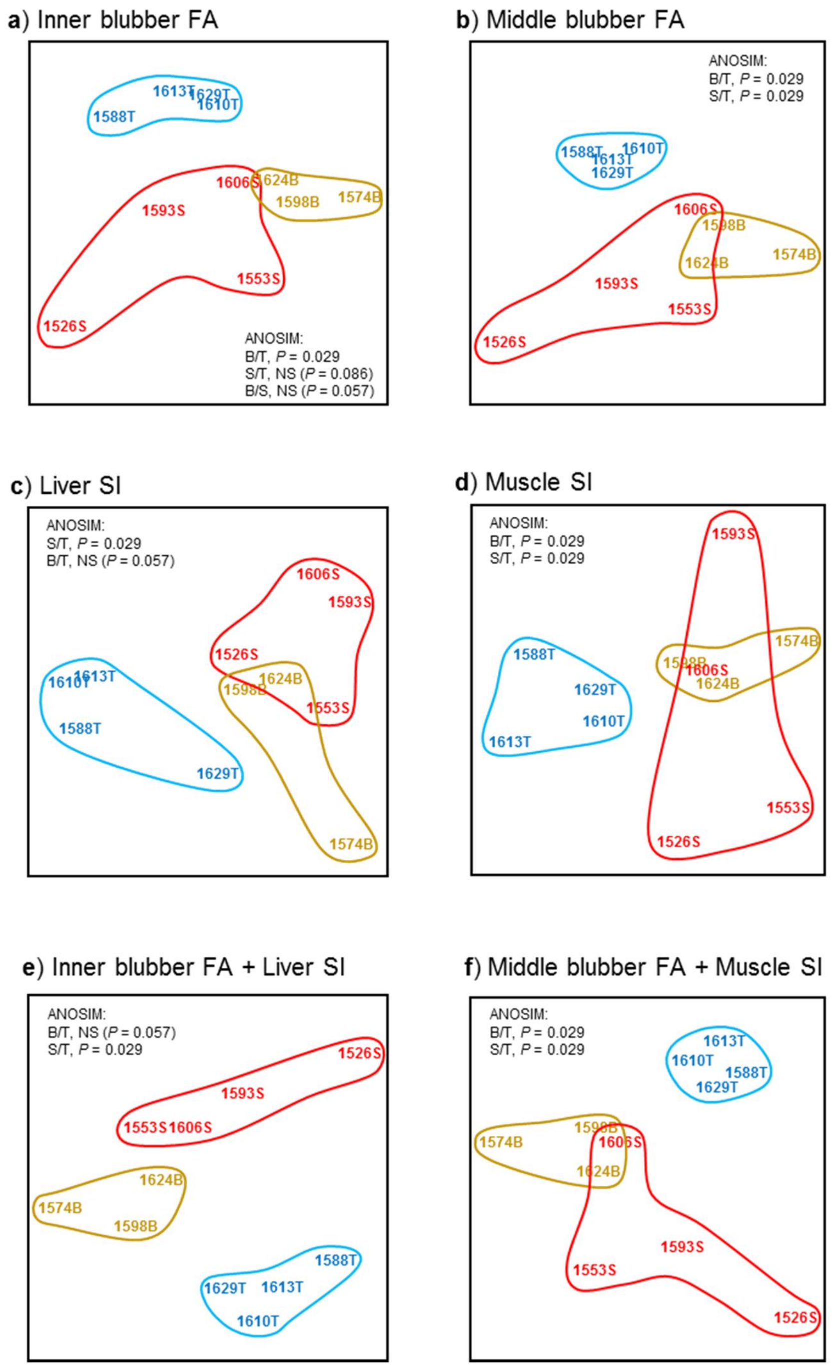

Fig. MDS plots of **a**) inner blubber FA, **b**) middle blubber FA, **c**) liver SI, **d**) muscle SI, **e**) combined inner blubber FA and liver SI data, and **f**) combined middle blubber FA and muscle SI data for 11 Finnish adult male grey seal individuals. The ANOSIM results of the statistical significance of the compositional differences are listed in the corners of the plots. T = bycaught in trawl, S = bycaught in surface fyke, B = bycaught in bottom fyke.
